# Actin Polymerization Defects Induce Mitochondrial Dysfunction in Cellular Models of Nemaline Myopathies

**DOI:** 10.3390/antiox12122023

**Published:** 2023-11-21

**Authors:** Rocío Piñero-Pérez, Alejandra López-Cabrera, Mónica Álvarez-Córdoba, Paula Cilleros-Holgado, Marta Talaverón-Rey, Alejandra Suárez-Carrillo, Manuel Munuera-Cabeza, David Gómez-Fernández, Diana Reche-López, Ana Romero-González, José Manuel Romero-Domínguez, Rocío M. de Pablos, José A. Sánchez-Alcázar

**Affiliations:** 1 Departamento de Fisiología, Anatomía y Biología Celular, Centro Andaluz de Biología del Desarrollo (CABD-CSIC-Universidad Pablo de Olavide), 41013 Sevilla, Spain; rpieper@alu.upo.es (R.P.-P.); alopcab2@alu.upo.es (A.L.-C.); malvcor@upo.es (M.Á.-C.); pcilhol@alu.upo.es (P.C.-H.); mtalrey1@alu.upo.es (M.T.-R.); asuacar1@alu.upo.es (A.S.-C.); mmuncab@upo.es (M.M.-C.); dgomfer1@acu.upo.es (D.G.-F.); dreclop@alu.upo.es (D.R.-L.); aromgon1@upo.es (A.R.-G.); jmromdom@upo.es (J.M.R.-D.); 2 Departamento de Bioquímica y Biología Molecular, Facultad de Farmacia, Universidad de Sevilla, 41012 Sevilla, Spain; depablos@us.es; 3 Instituto of Biomedicina de Sevilla (IBiS), Hospital Universitario Virgen del Rocío (HUVR)/CSIC/Universidad de Sevilla, 41012 Sevilla, Spain

**Keywords:** nemaline myopathy, actin polymerization, mitochondria, linoleic acid, L-carnitine

## Abstract

Nemaline myopathy (NM) is one of the most common forms of congenital myopathy and it is identified by the presence of “nemaline bodies” (rods) in muscle fibers by histopathological examination. The most common forms of NM are caused by mutations in the *Actin Alpha 1* (*ACTA1*) and *Nebulin* (*NEB*) genes. Clinical features include hypotonia and muscle weakness. Unfortunately, there is no curative treatment and the pathogenetic mechanisms remain unclear. In this manuscript, we examined the pathophysiological alterations in NM using dermal fibroblasts derived from patients with mutations in *ACTA1* and *NEB* genes. Patients’ fibroblasts were stained with rhodamine–phalloidin to analyze the polymerization of actin filaments by fluorescence microscopy. We found that patients’ fibroblasts showed incorrect actin filament polymerization compared to control fibroblasts. Actin filament polymerization defects were associated with mitochondrial dysfunction. Furthermore, we identified two mitochondrial-boosting compounds, linoleic acid (LA) and L-carnitine (LCAR), that improved the formation of actin filaments in mutant fibroblasts and corrected mitochondrial bioenergetics. Our results indicate that cellular models can be useful to study the pathophysiological mechanisms involved in NM and to find new potential therapies. Furthermore, targeting mitochondrial dysfunction with LA and LCAR can revert the pathological alterations in NM cellular models.

## 1. Introduction

Congenital myopathies are a group of genetic muscle diseases that are classified based on the histopathological features observed on muscle biopsy [1]. Nematine myopathy (NM) is a congenital musculoskeletal condition characterized by the presence of inclusions in muscle fibers known as “nemaline rods” [2]. This pathology contains a wide genetic heterogeneity, since multiple mutations in different genes that produce a similar phenotype have been identified [3]. Its incidence is approximately 1 in 50,000 live births.

The International European Neuromuscular Group classifies NM into six clinical subtypes based on the severity, age of onset, and degree of muscle weakness: severe congenital (neonatal), Amish, intermediate congenital, typical congenital, childhood onset, and adult onset [4]; of these, the most common subtype corresponds to the typical congenital subtype [5]. The most typical clinical manifestation is defined by a generalized weakness or hypotonia that mostly affects the proximal limb, axial and facial muscles, and that begins in early infancy or childhood. Additional features include skeletal deformities, dysmorphic fascia, arched high palate, and respiratory distress with respiratory tract infections [1]. The natural history of the disease is usually static or very slowly progressive and many of the patients can lead normal lives. Serious clinical complications are almost always secondary to respiratory deficiency and sometimes to cardiological problems. In most cases, cardiomyopathy develops in adulthood, while it rarely occurs in childhood [6].

There are 14 known causative genes of NM [7]. Mutations in the *ACTA1* and *NEB* genes, which code for actin alpha 1 (ACTA1) and nebulin (NEB) proteins, respectively, critical components of the sarcomeric thin filament, result in the most prevalent types of NM. More specifically, it is predicted that mutations in the *NEB* gene account for over 50% of cases of NM, whereas mutations in the *ACTA1* gene account for 15–25% of cases [8]. NEB, known as the giant actin-binding protein because it has a molecular weight of 600–800 kDa, plays a very important role in stabilizing and regulating the length of the actin filament [9]. ACTA1 is the actin isoform predominantly found in the thin filaments of skeletal muscles and essential, together with myosin, for muscle contraction [10].

The diagnosis is established from the clinical suspicion, since paraclinical studies such as creatinine kinase levels may be normal or slightly elevated and electromyography may show non-specific myopathic changes. The diagnosis is confirmed histopathologically by muscle biopsy analysis, in which characteristic rod bodies (nemaline bodies) are found in the sarcoplasm, evidenced by modified Gomori stain [1]. Currently, due to advances in genetic tools, it is increasingly common for the diagnosis to be made or confirmed by molecular genetic study for mutations in the genes known to cause the disease. Rods have been also detected in different cell culture lines, such as NIH3T3 fibroblasts or C2C12 myoblasts [11,12,13,14]. Specifically, both Costa et al. and Vandamme et al. demonstrated that NIH3T3 fibroblasts transfected with NM mutations presented cellular defects typical of the disease such as cytoplasmic actin aggregates and the formation of nemaline bodies or rods that are observed in patient skeletal muscle fibers [12,14].

The etiology of the disease remains unclear. The accumulation of nemaline bodies by itself does not explain the muscle weakness characteristic of the disease and could be only a secondary phenomenon of the main pathogenic process. In fact, no correlation has been observed between the severity of the disease and the degree of accumulation of nemaline bodies [12]. In addition to nemaline bodies, pathological defects have also been observed in skeletal muscle fibers both in mouse models and in patients with NM: abundant, unevenly spaced, and with irregular morphology nuclei; interrupted nuclear envelope; impaired chromatin arrangement; and cytoskeletal disorganization [15]. Due to the important function of the nuclear form and the envelope in the control of gene expression, and of the cytoskeleton in preserving the integrity and stability of the muscle fiber, it is likely that these alterations are responsible for some distinctive features of the disease, such as the disorder of the contractile filaments and the altered mechanical properties.

The disease has also been associated with mitochondrial dysfunction, specifically, with complex I dysfunction or deficiency [16]. One hypothesis proposes that by disrupting the integrity of the thin filaments and, consequently, disrupting normal muscle function, less energy is used and there is a “downregulation” of complex I activity and, therefore, adenosine triphosphate (ATP) production [16]. In addition, regarding the formation of nemaline rods, a high number of rods were found in some patients with complex I deficiency [17]. It has been seen that the formation of these can be induced in muscle and non-muscle cells in vitro by several different cellular stressors [18]. These data suggest that metabolic alterations during the formation and turnover of sarcomeres may induce the formation of nemaline rods. However, it is still unclear how these two pathological phenomena are related. Furthermore, interactions between mitochondria and the actin cytoskeleton have been linked to essential functions of this organelle [19]. Thus, actin filaments primarily modulate mitochondrial dynamics [20,21], trafficking, and autophagy [22] but also mitochondrial biogenesis and metabolism [23]. Therefore, it is reasonable to deduce that the actin polymerization defects in NM such as *ACTA1* and *NEB* mutations may affect mitochondrial function.

In order to achieve a deeper understanding of the mechanisms behind the disease’s development, animal and cell models are needed. Currently, animal and in vitro models of NM are being explored, specifically mouse and zebrafish models [24]. Regarding cellular models, in addition to in vitro contraction studies of muscle fibers [25], functional studies have been conducted on specific genetic mutations and their proteins in several cell lines, such as NIH3T3 fibroblasts, C2C12 myoblasts, or Sol 8 myogenic cells that differentiate into myotubes [13,26]. Most patient mutations studied by cell transfection reveal cellular defects typical of the disease, such as actin aggregates and the formation of nemaline bodies seen in patients’ muscle biopsies. On the other hand, regarding human cell lines of the disease, recently, two isogenic lines of induced pluripotent stem cells (iPSCs) derived from a patient with severe nemaline myopathy with a dominant heterozygous mutation in the *ACTA1* gene have been achieved [27]. Alternatively, patient-derived skin fibroblasts can be easily obtained by small biopsies of the skin in a non-invasive way, have a great capacity for division, and harbor the specific mutations of the patients [28].

In this manuscript, we have evaluated whether fibroblasts derived from NM patients carrying *ACTA1* and *NEB* mutations can be useful cellular models for studying disease pathophysiology. The work is based on the hypothesis that both affected proteins, ACTA1 and NEB, may participate in the formation and stabilization of actin filaments and, therefore, alterations of actin polymerization can be visualized in patient-derived fibroblasts. In addition, we examined the consequences of actin polymerization defects on mitochondrial function. Finally, we also evaluated the correction of all pathological alterations by mitochondrial-targeting compounds such as linoleic acid (LA) and L-carnitine (LCAR).

## 2. Materials and Methods

### 2.1. Reagents

The following antibodies were acquired from Abcam (Cambridge, United Kingdom): alpha skeletal muscle actin (ab28052), alpha tubulin (ab7291), NADH:ubiquinone oxidoreductase subunit A9 (NDUFA9) (ab14713), cytochrome c oxidase subunit IV (COXIV) (ab14744), ATP synthase F1 subunit alpha (ATP5F1A) (ab1478), voltage-dependent anion channel 1 (VDAC1) (ab14734), and superoxide dismutase 2 (SOD2) (ab68155). The following antibodies were purchased from Invitrogen (Thermo Fisher Scientific (Whaltham, MA, USA)): Rho (A, B, C) (PAI-338), Rho-associated protein kinase 1 (ROCK1) (PA5-22262), phospho-ROCK1 (PA5-36763), and NADH:ubiquinone oxidoreductase core subunit S1 (NDUFS1) (PA5-22309). RhoA (8789S), vimentin (D21H3), and glutathione peroxidase 4 (GPX4) (52455S) were purchased from Cell Signaling (Danvers, MA, USA). Antibodies for beta actin (MBS448085) and mitochondrially encoded NADH:ubiquinone oxidoreductase core subunit 1 (Mt-ND1) (6888S) were purchased from MyBioSource (San Diego, CA, USA). Cytochrome b-c1 complex subunit 2 (UQCR2) was supplied from US Biological (Salem, MA, USA). The following antibodies were acquired from Santa Cruz Biotechnology (Santa Cruz, CA, USA): dynamin-related protein 1 (DRP1) (sc-32898), succinate dehydrogenase complex iron–sulfur subunit B (SDHB) (sc-271548), and superoxide dismutase 1 (SOD1) (sc-101523). Optic atrophy type 1 (OPA1) (HPA036926) was acquired from Sigma-Aldrich (San Luis, MO, USA). Novus Biologicals (Centennial, CO, USA) provided mitochondrially encoded cytochrome c oxidase subunit 2 (Mt-CO2) (NBP1-778220). Trypsin, dimethyl sulfoxide (DMSO), saponin, Tris base, 4′,6-diamidino-2-phenylindole (DAPI), tetramethylethylenediamine (TEMED), Dulbecco’s modified Eagle’s medium (DMEM) 4.5 g/L and 1 g/L glucose, L-glutamine, pyruvate (Gibco), penicillin:streptomycin 10,000:10,000 (Gibco), fetal bovine serum (FBS) (Gibco), Mowiol 4-88 Mw (Sigma Chemical Co. (St. Louis, MO, USA)) were used. Bovine serum albumin (BSA) (Santa Cruz Biotechnology (Santa Cruz, CA, USA)) was used. A PierceTM BCA Protein Assay Kit (Fisher Scientific, Waltham, MA, USA) was used. A Rho Activation Assay Kit (Rf. 8820) (Cell Signaling Technology (Danvers, MA, USA)) was used. Rhodamine–phalloidin reagent (Abcam (Cambridge, UK)) was used. Acrylamide 37.5:1 solution, Clarity TM Western ECL substrate, electrophoresis buffer (TGS), sodium dodecyl sulfate (SDS), Triton X-100, blot buffer (TG), Tween 20, DC Protein Assay Reagent A, B, S (Bio-Rad Laboratories Inc. (Hercules, CA, USA)) were used. 2-Isopropanol, ethanol, methanol, sodium chloride (NaCl), ammonium persulfate (APS), glacial acetic acid, potassium hydroxide (KOH), potassium chloride (KCL) (Panreac (Barcelona, Spain)), and Protease Inhibitor Cocktail (Roche (F. Hoffmann-La Roche Ltd., Basel, Switzerland)) were used. L-carnitine (sc-205727), Y27632 (sc-3536), and paraformaldehyde (PFA) (sc-25326B) were purchased from Santa Cruz Biotechnology (Santa Cruz, CA, USA). Sigma-Aldrich (San Luis, MO, USA) supplied linoleic acid (L1376).

### 2.2. Patients and Cell Culture

Four cell lines of fibroblasts derived from skin biopsies of patients from the Pediatric Department of Hospital Universitario Virgen del Rocío, Sevilla, Spain were used. Fibroblast cell lines were isolated according to a previously described protocol [29]. Two control lines of primary human skin fibroblasts were acquired from ATCC. Patient 1 (P1) presents a heterozygous pathogenic variant c.133G > T (p.Val45Phe) in *ACTA1* that results in a missense variant. The second patient (P2) is also heterozygous with changes in position c.760A > T (p.Asn254Tyr) in the *ACTA1* gene. The third patient (P3) presents heterozygous pathogenic variants c.10321A > C (p.Thr3441Pro) and c.13669C > T (pArg4557*) in *NEB*. The fourth patient (P4) presents heterozygous pathogenic variants c.24407_24410dup (p.Leu8137Phefs*18) and c.8425C > T (p.Arg2809*) in *NEB*.

Control values were represented as mean ± SD of two control lines. Fibroblasts were grown in Dulbecco’s modified Eagle’s medium (DMEM) (Gibco™, ThermoFisher Scientific, Waltham, MA, USA) supplemented with 10% FBS (Gibco™, ThermoFisher Scientific, Waltham, MA, USA) and 100 mg/mL penicillin/streptomycin. Fibroblasts were cultured under conditions of 37 °C and 5% CO_2_. Experiments were performed with less than 12-passage fibroblast cultures.

### 2.3. Immunoblotting

Standard procedures were followed to conduct the Western blotting [30]. Subsequently protein transfer, the membrane was incubated with various primary antibodies diluted 1:1000 and then with the corresponding secondary antibody coupled to horseradish peroxidase (HRP) at a 1:10,000 dilution. The Immun-Star HRP substrate kit (Biorad Laboratories Inc., Hercules, CA, USA) was used to identify particular proteins.

### 2.4. Real-Time Quantitative PCR (qPCR)

*ACTA1* and *NEB* gene expression in fibroblasts was analyzed by qPCR using mRNA extracts. Using TrizolTM (Invitrogen, Carlsbad, CA, USA), mRNA was extracted in accordance with the manufacturer’s instructions. To obtain complementary DNA (cDNA), the Iscript cDNA synthesis Kit (Bio-Rad, Hercules, CA, USA) was used to retrotranscribe RNA. TB Green^TM^ Premix Ex Taq^TM^ (Takara Bio Europe S.A.S., Saint-Germain-en-Laye, France) was used for qPCR. To accurately quantify gene expression, a CFX Connect Real-Time PCR Detection System (Bio-Rad, Hercules, CA, USA) was used. The forward and reverse primers for ACTA1 were 5′-CCATTTATGAGGGCTACGCG-3′ and 5′-CAGCTTCTCCTTGATGTCGC-3′, respectively, each of which amplified a 158-nucleotide sequence. The forward and reverse NEB primers were 5′-GAAACCAGACCACAGCCTTG-3′ and 5′-TAGGGCATCTTTCACCGTGT-3′, respectively, amplifying a 224-nucleotide sequence. As a housekeeping control gene, human *α-Tubulin* was used and the primers were 5′-GCAGCATTTGTAGCAGGTGA-3′ (forward primer) and 3′-GCATTGCCAATCTGGACAC-5′ (reverse primer). Prior to gel electrophoresis, PCR was used to validate each primer pair, ensuring that the correct product was amplified.

### 2.5. Active RhoA Assay

Fibroblasts were grown in 75 cm^2^ flasks in DMEM containing 10% FBS. According to the manufacturer’s recommendations, media were removed, cells were washed twice using ice-cold PBS 1×, and the cell lysate was collected using lysis/binding/wash buffer supplemented with a 1x protease inhibitor cocktail provided by the Active Rho Detection Kit (Cell Signaling Technology, Danvers, MA, USA, Cat. 8820). After centrifuging at 16,000× *g* at 4 °C for 15 min, lysate protein concentration was determined using a BCA Protein Assay Kit and 1 mg/mL lysate in 1x lysis/binding/wash buffer was prepared. After making the cell lysate stock, protein then was incubated with GST Rhotekin-RBD affinity beads at 4 °C for 1 h with gentle rocking. Bound proteins (active RhoA form) were washed and prepared with SDS Sample Buffer and then Western blotting analysis was carried out according to the previously mentioned protocol.

### 2.6. Immunofluorescence Microscopy

Fibroblasts were cultured on 1 mm wide glass coverslips (Goldseal No. 1) for 24–48h in DMEM supplemented with 20% FBS. Cells were rinsed once with PBS 1×, fixed in 4% PFA for five minutes at room temperature, and permeabilized for ten minutes in 0.1% saponin. Then, fibroblasts were blocked with blocking solution (1% BSA in PBS 1×). For immunostaining, glass coverslips were incubated with primary antibodies diluted 1:100 and prepared in blocking solution overnight at 4 °C. Unbound antibodies were removed by washing the coverslips with PBS 1× (three times, for five minutes). The secondary antibody, a FITC-labeled goat anti-mouse antibody or tetramethyl rhodamine goat anti-rabbit (Molecular Probes), diluted 1:400 and prepared in blocking solution, was added and incubated for two hours at room temperature. Coverslips were then rinsed with PBS 1× for five minutes, incubated for one minute with PBS 1× containing DAPI (1 μg/mL), and washed with PBS 1× (three five-minute washes). Finally, the coverslips were mounted onto microscope slides using Mowiol aqueous mounting medium. Samples were analyzed using a DeltaVision system (Applied Precision; Issaquah, WA, USA) with an Olympus IX-71 microscope (Olympus Corporation, Shinjuku, Tokyo, Japan). Colocalization was analyzed by Pearson correlation coefficient calculated with the DeltaVision system.

### 2.7. Immunofluorescence Staining of Cytoskeletal F-Actin

Briefly, fibroblasts were seeded on 1 mm wide coverslips (Goldseal No. 1) for 24–48 h in DMEM supplemented with 10% FBS. Next day, after reaching to 50% confluency, cells were fixed with 4% PFA for 10 min at room temperature and permeabilized with saponin at 0.1% for 15 min. Then, cells were washed with PBS 1× twice and incubated with rhodamine–phalloidin, a high-affinity F-actin probe conjugated with a red-orange fluorescent dye, at a concentration of 1 μg/mL for 30 min. After incubation, cells were washed with PBS 1× three times and incubated with DAPI at 1 μg/mL for 5 min for nuclei staining. Subsequently, cells were washed three times with PBS 1× for 5 min. Finally, the coverslips were mounted on microscope slides with Mowiol aqueous mounting medium. After staining, images were acquired by a DeltaVision system with an Olympus IX-71 fluorescence microscope using a 40× oil objective. The analysis of images was carried out by Fiji-ImageJ software (version 2.9.0/1.53t).

In order to evaluate the state of actin filaments, the percentage of cells with correct actin polymerization and the length of actin filaments present in each cell were examined. To estimate the percentage of cells with correct actin polymerization for each patient, three counts of 100 cells per sample were performed; the values of these counts were compared among themselves and between the different samples. Cells with correct actin polymerization were considered those cells that presented actin filaments similar to those presented by control cells. The lengths of actin filaments present in each cell were measured by Fiji-ImageJ software. Specifically, actin filament length measurements were made using the software’s options “Set scale” and “Measure”. The first option allows configuration of the exact scale according to the objective with which images were taken, ensuring that the relationship between pixel size and micrometers is correct. The option “Measure” works like a ruler and allows determination of the length in micrometers of any structures.

To execute this, the filaments of 30 cells of each cell line were measured in triplicate. The actin filament length values represented in the figures are expressed as the average of the measured filament lengths.

### 2.8. Bioenergetics and Oxidative Stress Analysis

Mitochondrial respiratory function of control and NM fibroblasts was determined using a mitostress test assay by an XF24 extracellular flux analyzer (Seahorse Bioscience, Billerica, MA, USA). Cells were cultured at a density of 15,000 cells/well in XF24 cell culture plates and in combination with 150 μL of growth medium (DMEM supplemented with 20% FBS) under conditions of 37 °C and 5% CO_2_. After 24 h of incubation, growth medium from each well was discarded, leaving only 50 μL of media. After that, 450 μL of assay medium (500 μL total) was added to each well after cells had been washed twice with 1 mL of pre-warmed assay medium (XF base medium supplemented with 10 mM glucose, 1 mM glutamine, and 1 mM sodium pyruvate; pH 7.4). For one hour, fibroblasts were incubated in a 37 °C incubator without CO_2_ to allow pre-equilibrating with the assay medium. Mitochondrial functionality was examined by sequential injection of four substances that affect bioenergetics. The four compounds were injected at the following final concentrations: 2 μM FCCP (carbonyl cyanide-4-trifluoromethoxy-phenylhydrazone), 1 μM, oligomycin, and 2.5 μM antimycin A/rotenone. Preliminary experiments were conducted to determine the optimal cell seeding density, as well as the best concentration of each inhibitor and uncoupler. In each experiment, a minimum of five wells were used per treatment. This assay allowed for an estimation of important mitochondrial parameters such as basal and maximal respiration, ATP production, and spare respiratory capacity. Results obtained by the XF24 analyzer were normalized based on the number of cells seeded (15,000 cells). To check if the number of cells was still stable, cell counting of each well was performed using the BioTek^TM^ Cytation^TM^ 1 Cell Imaging Multi-Mode Reader before and after the assay.

### 2.9. Mitotracker Staining: Analysis of Mitochondrial Network

Mitochondrial membrane potential (ΔΨm) was assessed in fibroblasts by fluorescence microscopy analyzing the fluorescence intensity derived from MitoTracker Red CMXRos staining (100 nM, 45 min, 37 °C) by Fiji-ImageJ software. ΔΨm/fluorescence intensity was determined in 100 cells in three different experiments.

The degree of mitochondrial fragmentation was determined using the Fiji software. To determine the number of small, rounded mitochondria per cell, a total of 100 cells from three different experiments were used for each experimental condition. The percentage of rounded/tubular mitochondria, the degree of circularity, and the length or ratio between the major and minor axis of the mitochondrion were considered [31]. Specifically, high-quality 40× images were analyzed by Fiji-ImageJ software and a threshold image was applied to select only the mitochondrial network. To determine percentages of rounded/tubular mitochondria, the software’s option “Analyze particles” was used, analyzing size particles and circularity. For rounded mitochondria, a size of 0.2–0.9 was chosen. In the case of tubular mitochondria, a size of 0.9–infinity was chosen. For both determinations, images were acquired by a DeltaVision system with an Olympus IX-71 fluorescence microscope using 40×/60× oil objectives.

### 2.10. Measurement of Intracellular Reactive Oxygen Species (ROS) Generation

According with the manufacturer’s instructions, MitoSOX™ Red at 5 μM was used to measure the fibroblasts’ mitochondrial superoxide production. Previously, cells were grown on coverslips until reaching 80% confluency. Mitochondrial localization of MitoSOX™ Red signal was confirmed in conjunction with MitoTracker™ Deep Red FM staining (at 100 nM, 45 min, 37 °C), an in vivo mitochondrial membrane-potential-independent probe. Cells’ nuclei were stained with DAPI at 1 μg/mL. After staining, images were generated by a DeltaVision system with an Olympus IX-71 fluorescence microscope using a 40× oil objective. Images were analyzed by Fiji-ImageJ software.

### 2.11. Statistics

Statistical analysis was conducted in accordance with our research group’s previous description [27]. In situations where there were few events (*n* < 30), we employed non-parametric statistics that do not make any distributional assumptions [28]. In these cases, multiple groups were compared using a Kruskal–Wallis test. We used parametric tests when the number of events was greater (*n* > 30). In these instances, a one-way ANOVA was used to compare multiple groups. Statistical analyses were conducted using GraphPad Prism 9.2 (GraphPad Software, San Diego, CA, USA). The information is presented as the mean ± SD values or as an example from three independent experiments. *p*-values of less than 0.05 were considered significant.

## 3. Results

### 3.1. NM Fibroblasts Present Alterations in Actin Alpha 1 (ACTA1) and Nebulin (NEB) Expression Levels

**Figure 1:**
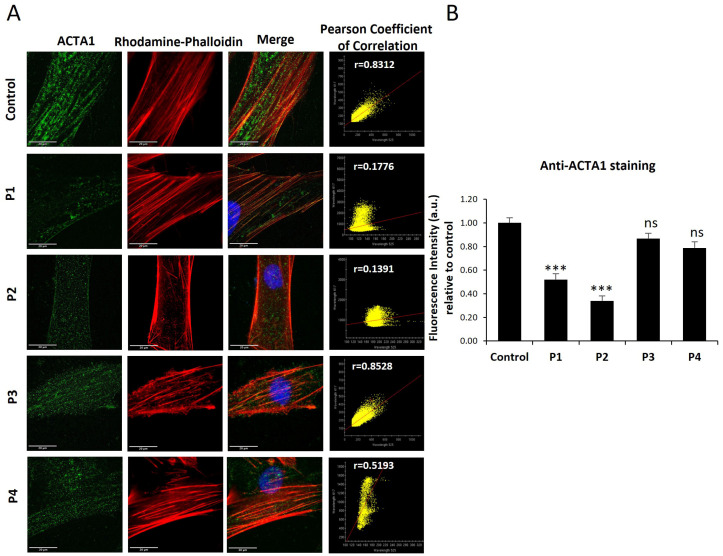
ACTA1 expression levels by immunofluorescence microscopy in control and NM cells. (**A**) Control (C1) and NM cells (P1, P2, P3, and P4) were immunostained against ACTA1 and visualized under a fluorescence microscope. Nuclei were revealed by DAPI staining. The colocalization between ACTA1 signal and F-actin staining by rhodamine–phalloidin was analyzed by Pearson correlation coefficient. Pearson correlation coefficient was calculated with a DeltaVision system. A positive correlation was considered when Pearson coefficient > 0.75. (**B**) Quantification of ACTA1 signal. Images were taken using a 60× lens and processed by ImageJ software. *** *p* < 0.001 between NM and control cells. Scale bars = 20 µm; n.s = no significant.

**Figure 2:**
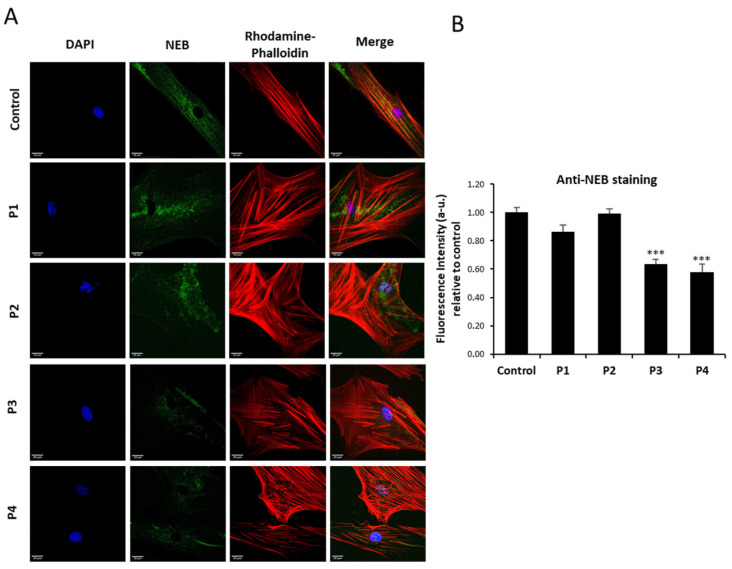
NEB expression levels by immunofluorescence microscopy in control and NM cells. (**A**) Control (C1) and NM cells (P1, P2, P3, and P4) were immunostained against NEB and visualized under a widefield fluorescence microscope. Nuclei were revealed by DAPI staining. (**B**) Quantification of NEB signal. Images were taken using a 40× lens and processed by ImageJ software. *** *p* < 0.001 between NM and controls cells. Scale bars = 20 µm.

First, we analyzed ACTA1 and NEB protein expression levels in control and NM-patient-derived fibroblasts (P1 and P2 harboring ACTA1 mutations; and P3 and P4 carrying NEB mutations). As shown by immunofluorescence microscopy in Figure 1A,B, ACTA1 expression levels were markedly reduced in P1 and P2 cells, while NEB (Figure 2A,B) expression levels were reduced in P3 and P4. Interestingly, ACTA1 immunostaining colocalized with actin filaments in control cells but not in mutant cells.

**Figure 3:**
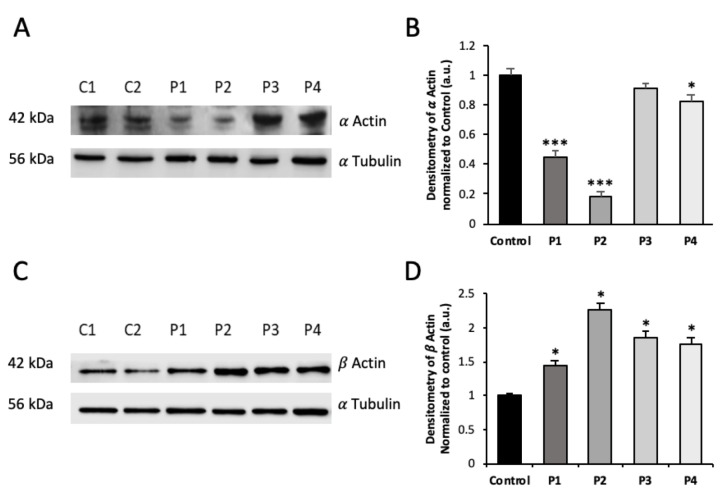
Protein expression levels of α-actin and β-actin NM cells. (**A**) Cellular extracts from controls (C1 and C2) and NM patient cell lines P1, P2, P3, and P4 were subjected to immunoblotting analysis. An SDS polyacrylamide gel was used to separate protein extracts (50 μg), then the samples were immunostained using antibodies against ACTA1 and α-tubulin, which was used as a loading control. (**B**) Densitometry of the Western blotting. (**C**) Cellular extracts from controls (C1 and C2) and NM patient cell lines P1, P2, P3, and P4 were subjected to immunoblotting analysis. An SDS polyacrylamide gel was used to separate protein extracts (50 μg), then the samples were immunostained using antibodies against β-actin and α-tubulin, which was used as a loading control. (**D**) Densitometry of the β-actin Western blotting. Data represent the mean ± SD of three separate experiments. * *p* < 0.05, *** *p* < 0.001 between NM cells and controls. a.u., arbitrary units.

**Figure 4:**
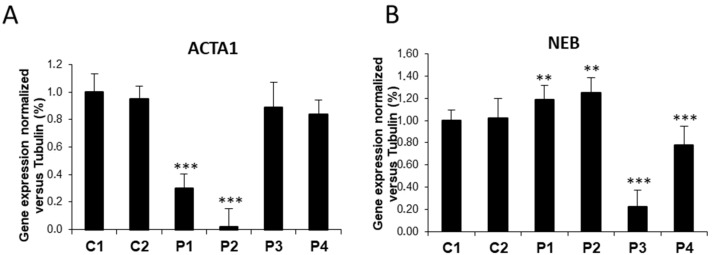
ACTA1 and NEB transcript expression levels in control and NM cells. (**A**) *ACTA1* gene expression quantified by RT-PCR. Results were normalized to *α-Tubulin* gene expression. (**B**) *NEB* gene expression quantified by RT-PCR. Results were normalized to *α-Tubulin* gene expression. Data represent the mean ± SD of three separate experiments. ** *p* < 0.01, *** *p* < 0.001 between NM cells and controls.

Reduced expression levels of ACTA1 were confirmed by Western blotting analysis (Figure 3A,B). Low protein expression levels of mutant proteins were associated with a reduction in *ACTA1* transcript levels in P1 and P2 mutant fibroblasts (Figure 4A). Interestingly, expression levels of β-actin (cytoplasmic actin isoform and component of microfilaments) were upregulated both in *ACTA1* and *NEB* mutant fibroblasts (Figure 3C,D).

Moreover, *NEB* transcripts levels in P3 and P4 were confirmed, suggesting a decrease in gene expression of affected genes or an increase in mutant transcript degradation in NM cells (Figure 4A,B). Remarkably, NEB transcripts levels in ACTA1 mutant cells (P1, P2) were significantly higher than the expression levels shown by control cells (** *p*-value < 0.01), suggesting that NEB overexpression may be acting as a compensation mechanism for defects in ACTA1 (Figure 4B).

### 3.2. NM-Patient-Derived Fibroblasts Show Defects in Actin Filament Polymerization

**Figure 5:**
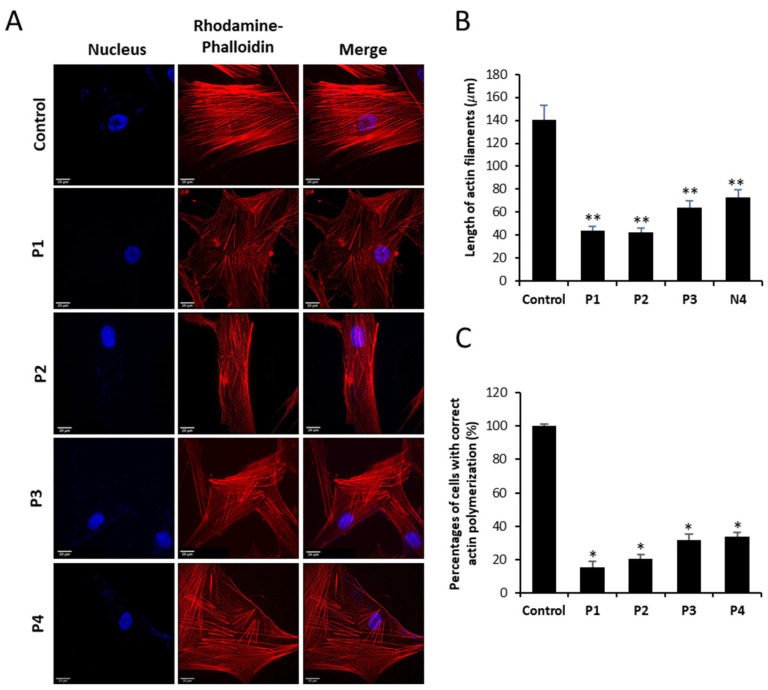
Actin staining by rhodamine–phalloidin of control and NM fibroblasts. (**A**) Control and NM fibroblasts, P1 and P2 (mutation in *ACTA1*) and P3 and P4 (mutation in *NEB*), were stained with rhodamine–phalloidin and visualized under a widefield fluorescence microscope. Nuclei were revealed by DAPI staining. NM fibroblasts (P1, P2, P3, and P4) presented smaller and unstructured actin filaments compared to control fibroblasts. Images were taken using a 40× lens and processed by ImageJ software. (**B**) Measurement of the length of actin filaments (μm). The length of the actin filaments was measured in triplicate with ImageJ software in 30 images. (**C**) Percentage of cells with correct actin polymerization. Three counts of 100 cells per sample were performed. * *p* < 0.05, ** *p* < 0.01 between NM and controls cells. Scale bar = 20 µm.

Cell staining with rhodamine–phalloidin showed that fibroblasts derived from NM patients have marked defects in the polymerization of actin filaments. Thus, mutant fibroblasts of P1 and P2 harboring *ACTA1* mutations and P3 and P4 harboring *NEB* mutations displayed unstructured actin filaments of shorter length than control fibroblasts (Figure 5A,B). In addition, the analysis revealed that the percentage of NM fibroblasts with correct actin filament polymerization was much lower than in control cells (Figure 5C). P1 fibroblasts showed a more severe defect in actin filament polymerization (only 7% of P1 fibroblasts showed a correct actin polymerization).

NM-patient-derived fibroblasts did not show alterations in the level of expression of other cytoskeletal proteins. Thus, the analysis of expression of vimentin (component of the intermediate filaments) and α-tubulin (component of microtubules) did not reveal significant differences in mutant fibroblasts compared to control fibroblasts (Supplementary Figure S1A,B).

### 3.3. RhoA/ROCK Pathway Is Overactivated in NM Fibroblasts

**Figure 6:**
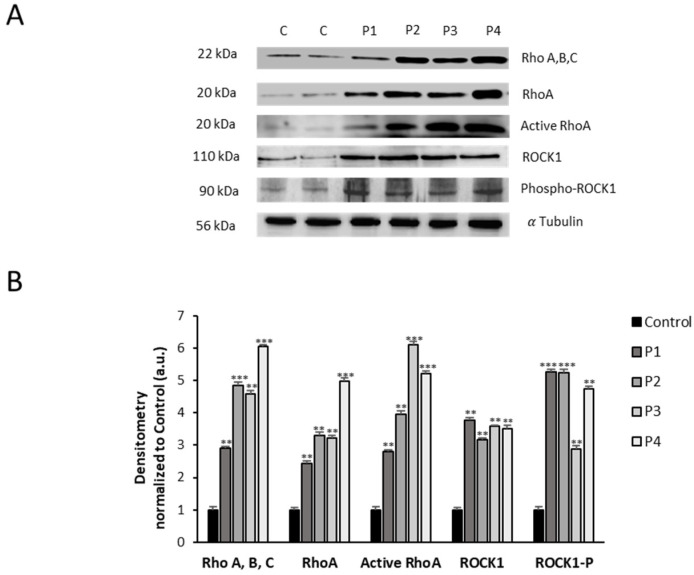
Expression level of RhoA/ROCK pathway in NM cells. (**A**) Cellular extracts from controls (C1 and C2) and NM patient cell lines P1, P2, P3, and P4 were subjected to immunoblotting analysis. An SDS polyacrylamide gel was used to separate protein extracts (50 μg), then the samples were immunostained using antibodies against Rho (A, B, C), RhoA, active RhoA purified by the Active Rho Detection Kit as described in the Material and Methods, ROCK1, phospho-ROCK1, and α-tubulin, which was used as a loading control. (**B**) Densitometry of the Western blotting. For control cells (C1 and C2), data are the mean ± SD of the two control cell lines. Data represent the mean ± SD of three separate experiments. ** *p* < 0.01, *** *p* < 0.001 between NM and controls cells. a.u., arbitrary units.

As the RhoA/ROCK pathway plays an essential role in actin polymerization [32,33], we next examined the expression levels of RhoA, active RhoA, ROCK1, and phosphorylated ROCK1. NM mutant cells P1, P2, P3, and P4 showed increased activation of active RhoA and phosphorylated ROCK1, indicating overactivation of the RhoA/ROCK pathway (Figure 6A,B). Interestingly, control cells treated with Y-27632, a ROCK inhibitor, mimicked the actin polymerization defects found in NM cells (Supplementary Figure S2A,B), suggesting that RhoA/ROCK pathway overactivation is a compensatory mechanism to increase actin polymerization in *ACTA1* and *NEB* mutations.

### 3.4. NM Fibroblasts Display Alterations in Mitochondrial Bioenergetics and Network Morphology

**Figure 7:**
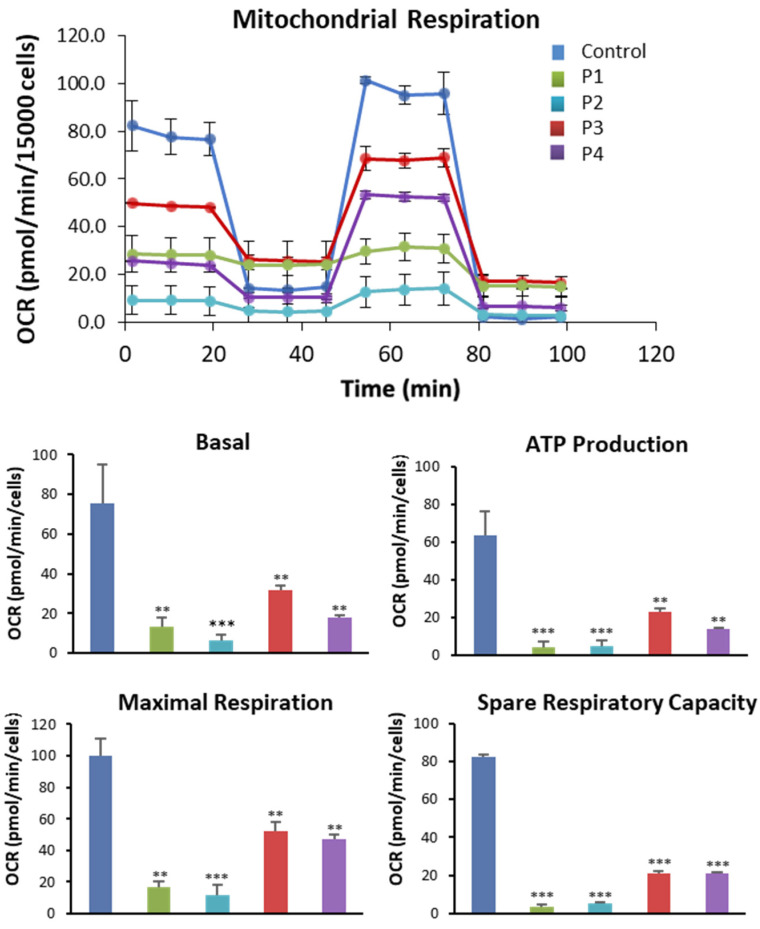
Bioenergetics analysis of control and NM fibroblasts. NM fibroblasts (P1, P2, P3, and P4) and controls (C1 and C2) were examined for basal and maximum respiration, mitochondrial ATP generation, and spare respiratory capacity measured using the Seahorse analyzer. The results were standardized to 15,000 cells according to the instructions in the Material and Methods. ** *p* < 0.01, *** *p* < 0.001 between NM and controls cells.

Since the actin cytoskeleton and the correct actin filament polymerization are essential for mitochondrial function [16], we further investigated mitochondrial bioenergetics in NM fibroblasts. For that purpose, mitochondrial bioenergetics parameters were examined in control and NM fibroblasts (Figure 7). Compared to control cells, fibroblasts derived from patients P1, P2, P3, and P4 had decreased basal, maximum, and spare respiration as well as lower mitochondrial ATP generation. These findings indicated that mutant fibroblasts showed a significant mitochondrial dysfunction.

**Figure 8:**
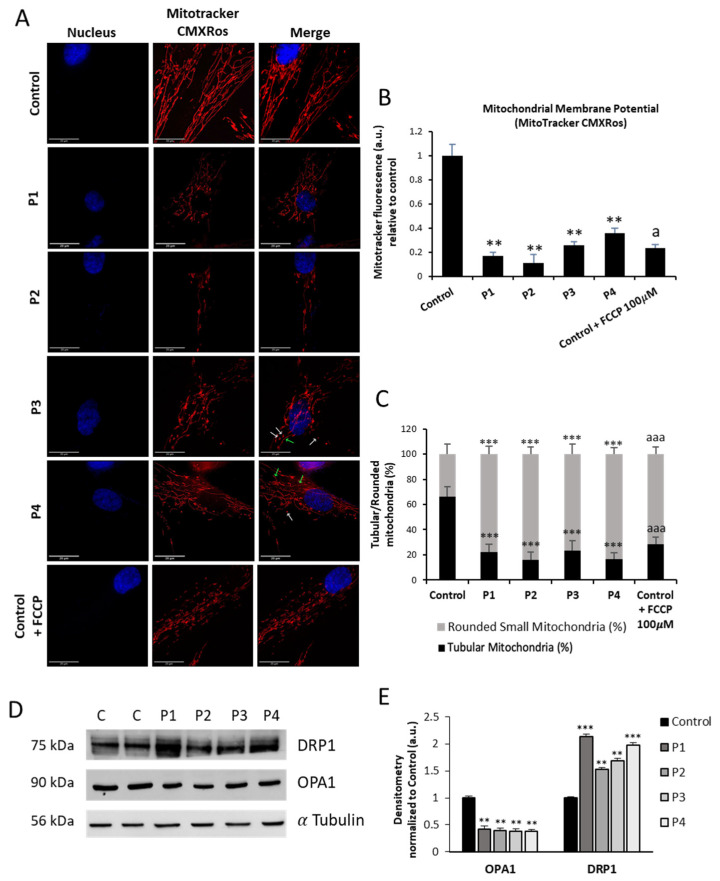
Mitochondrial polarization and network in control and NM cells. (**A**) Representative images of control (C1) and NM fibroblasts (P1, P2, P3, and P4) stained with MitoTracker^TM^ Red CMXRos and visualized under a widefield fluorescence microscope. Nuclei were revealed by DAPI staining; 100 µM CCCP was used for 4 h as a positive control of mitochondrial depolarization in control cells. Rounded small mitochondria are marked with white arrows and tubular mitochondria are marked with green arrows in P3 and P4. Images were taken using a 100× lens and processed by ImageJ software. Scale bar = 20 µm. (**B**) Fluorescence quantification of MitoTracker signal. Data represent the mean ± SD of three separate experiments (at least 100 cells for each condition and experiment were analyzed). (**C**) Quantification of tubular and rounded percentages of mitochondria in control and NM fibroblasts. Data represent the mean ± SD of three separate experiments (at least 100 cells for each condition and experiment were analyzed). (**D**) Cellular extracts from controls (C1 and C2) and NM patient cell lines P1, P2, P3, and P4 were subjected to immunoblotting analysis. An SDS polyacrylamide gel was used to separate protein extracts (50 μg), then the samples were immunostained using antibodies against DRP1, OPA1, and α-tubulin, which was used as a loading control. (**E**) Densitometry of the Western blotting. For controls cells (C1 and C2), data are the mean ± SD of the two control cell lines. Data represent the mean ± SD of three separate experiments. ** *p* < 0.01, *** *p* < 0.001 between NM cells and controls; ^a^ *p* < 0.05, ^aaa^ *p* < 0.001 between the presence and the absence of CCCP. a.u.: arbitrary units.

After labeling mitochondria with MitotrackerTM Red CMXRos, we proceeded to investigate the morphology of the mitochondrial network by fluorescence microscopy. Figure 8A illustrates representative images of the mitochondrial shape. A total of 100 cells were quantified for each condition (Figure 8B). The mitochondrial network morphology of mutant fibroblasts (P1, P2, P3, and P4) showed the presence of depolarized and fragmented mitochondria. Therefore, mitochondrial fragmentation was evaluated calculating the percentage of rounded and tubular mitochondria. Comparing NM cells to control cells, we found a significant decrease in tubular mitochondria (Figure 8C). Carbonyl cyanide monochlorophenylhydrazon (CCCP), 100 µM, was applied to control cells for four hours as a positive control of mitochondrial depolarization.

As the actin cytoskeleton plays a fundamental role in the regulation of mitochondrial dynamics [19], we next evaluated the expression levels of DRP1 and OPA1, the main proteins involved in mitochondrial fission and fusion processes, respectively. NM mutant cells P1, P2, P3, and P4 showed increased expression levels of DRP1 and reduced levels of OPA1 in comparison with control cells, indicating an imbalance between mitochondrial fission and fusion processes (Figure 8D,E). These results agree with the fragmented mitochondrial network (Figure 8A) and the reduced percentages of tubular mitochondria and increased percentages of rounded mitochondria (Figure 8C) presented by NM cells compared to control cells.

**Figure 9:**
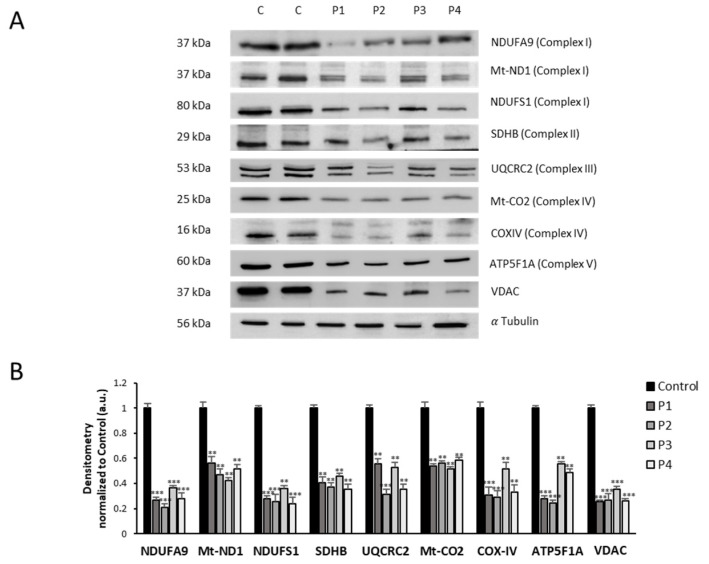
Mitochondrial protein expression levels in control and NM cells. (**A**) Cellular extracts from controls (C1 and C2) and NM patient cell lines P1, P2, P3, and P4 were subjected to immunoblotting analysis. An SDS polyacrylamide gel was used to separate protein extracts (50 μg), thens the samples were immunostained using antibodies against NDUFA9 (complex I), NDUFS4 (complex I), mtND1 (complex I), SDHB (complex II), UQCRC2 (complex III), mtCO2 (complex IV), COX4 (complex IV), ATP5A (complex V), and VDAC1. α-Tubulin was used as a loading control. (**B**) Densitometry of the Western blotting. For controls cells (C1 and C2), data are the mean ± SD of the two control cell lines. Data represent the mean ± SD of three separate experiments. ** *p* < 0.01, *** *p* < 0.001 between NM and controls cells. a.u., arbitrary units.

Mitochondrial dysfunction in mutant cells was also corroborated by examining mitochondrial protein expression levels (Figure 9A). Thus, expression levels of mitochondrial protein subunits NDUFA9 (complex I), NDUFS4 (complex I), mtND1 (complex I), SDHB (complex II), UQCRC2 (complex III), mtCOX2 (complex IV), COX4 (complex IV), ATP5A (complex V), and VDAC1 were significantly downregulated in NM mutant cells with respect to control cells.

### 3.5. NM-Patient-Derived Fibroblasts Present Increased ROS and Alterations in Antioxidant Enzyme Expression Levels

**Figure 10:**
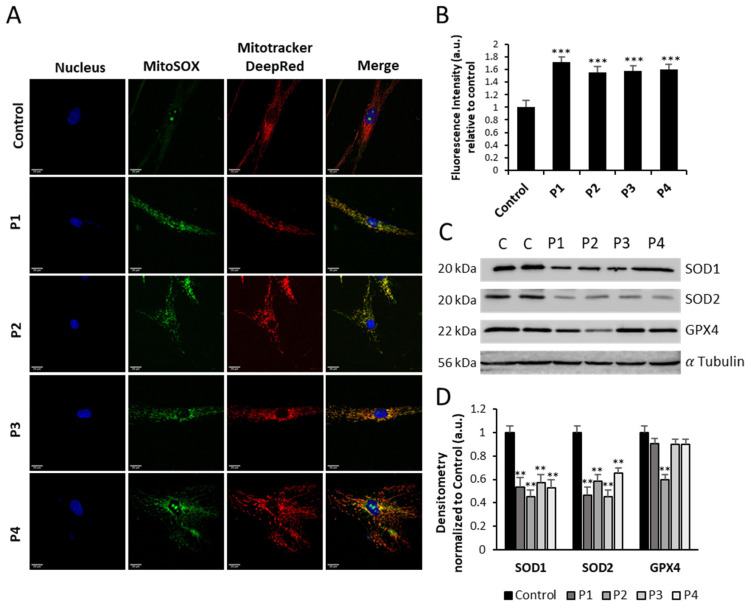
Expression levels of ROS and antioxidant enzymes in NM cells. (**A**) Representative images of control (C1) and NM fibroblasts (P1, P2, P3, and P4) stained with MitoSOX™ Red and MitoTracker^TM^ DeepRed. Nuclei were revealed by DAPI staining. Images were taken under a widefield fluorescence microscope using a 40× lens and processed by ImageJ software. Scale bar = 20 µm. (**B**) Fluorescence quantification of MitoSOX™ Red signal. Data represent the mean ± SD of three separate experiments (at least 100 cells for each condition and experiment were analyzed). (**C**) Cellular extracts from controls (C1 and C2) and NM patient cell lines P1, P2, P3, and P4 were subjected to immunoblotting analysis. An SDS polyacrylamide gel was used to separate protein extracts (50 μg), then the samples were immunostained using antibodies against SOD1, SOD2, and GPX4. α-Tubulin was used as a loading control. (**D**) Densitometry of the Western blotting. For control cells (C1 and C2), data are the mean ± SD of the two control cell lines. Data represent the mean ± SD of three separate experiments. ** *p* < 0.01, *** *p* < 0.001 between NM and controls cells. a.u., arbitrary units.

As mitochondrial dysfunction is related to an increase in oxidative stress and reactive oxygen species (ROS) production, we also studied the mitochondrial ROS generation by MitoSOX™ and the protein expression levels of antioxidant enzymes. Thus, NM cells showed a significant mitochondrial ROS overproduction compared to control cells (Figure 10A,B). Furthermore, the protein expression levels of cytoplasmic superoxide dismutase (SOD1), mitochondrial superoxide dismutase (SOD2), and glutathione peroxidases (GPX4) were also reduced in NM fibroblasts, suggesting that the enzymatic antioxidant system is downregulated in mutant NM cells (Figure 10C,D).

### 3.6. Supplementation with Linoleic Acid (LA) and L-Carnitine (LCAR) Restores Actin Polymerization

**Figure 11:**
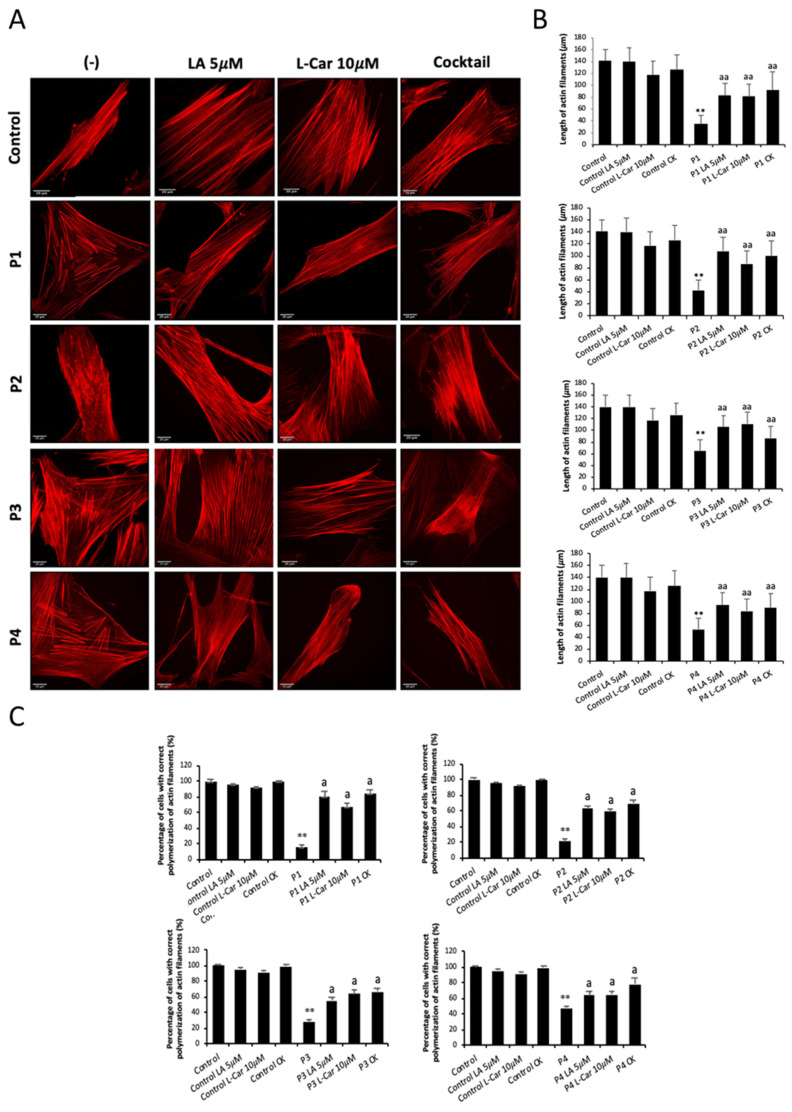
Effect of LA and LCAR on actin polymerization in control and NM cells. Control and NM fibroblasts were treated with 5 μM LA and 10 μM LCAR individually or in combination (Cocktail) for 7 days. (**A**) Representative images of treated and untreated (-) control and NM fibroblasts, P1 and P2 (mutation in *ACTA1*) and P3 and P4 (mutation in *NEB*) stained with rhodamine–phalloidin. Images were taken using a 40× lens and processed by ImageJ software. Scale bar = 20 µm. (**B**) Measurements of the length of actin filaments (μm). The length of the actin filaments was measured in triplicate with ImageJ software in 30 images. (**C**) Percentages of cells with correct actin polymerization. Three counts of 100 cells per sample were performed. ** *p* < 0.01 between NM and control cells; ^a^ *p* < 0.05, ^aa^ *p* < 0.01 between untreated (-) and treated NM cells.

After an initial pharmaceutical screening aiming to identify commercial supplements able to restore actin polymerization patterns in mutant cells, we selected two well-known mitochondrial-boosting compounds: LA and LCAR. Treatment with 5 µM LA and 10 µM LCAR for 7 days significantly improved actin filament polymerization in NM cells (Figure 11A). Thus, supplementation with both compounds separately and in combination significantly increased both the percentage of cells with correct actin polymerization and the length of the actin filaments in P1, P2, P3, and P4 fibroblasts (Figure 11B,C). LA and LCAR concentrations were chosen considering the efficiency in recovering actin polymerization in dose–response curve assays (Supplementary Figures S3–S6).

### 3.7. Supplementation with LA and LCAR Improves Mitochondrial Bioenergetics

**Figure 12:**
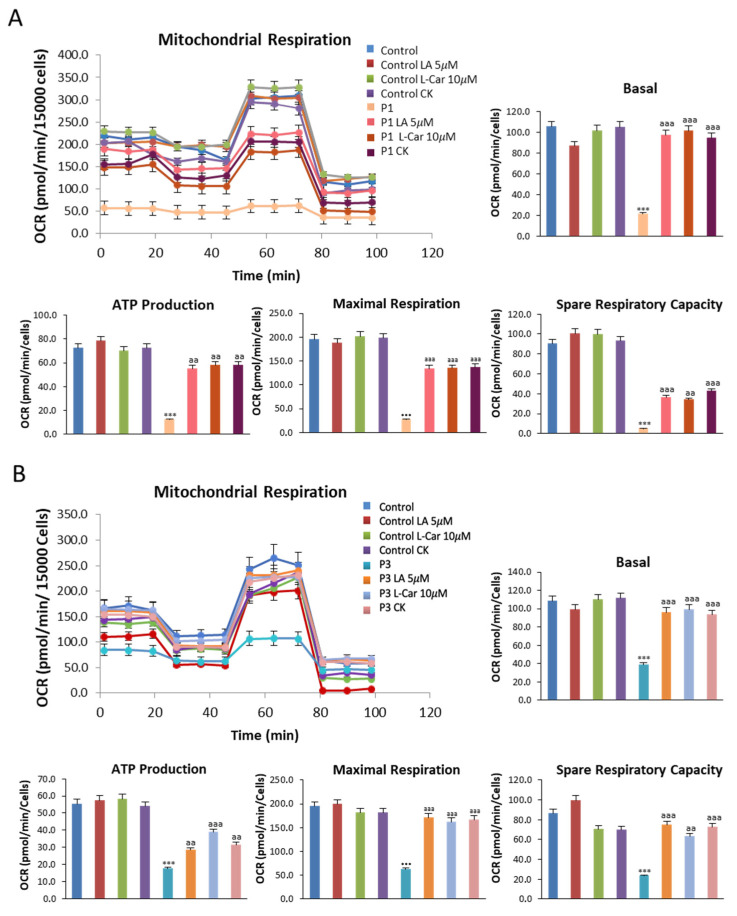
Effect of LA and LCAR on bioenergetics of control and NM fibroblasts. Control (C1) and NM fibroblasts (P1 and P3) were treated (+) with 5 μM LA and 10 μM LCAR individually or in combination for 7 days. Basal and maximal respiration, mitochondrial ATP production, and spare respiratory capacity were determined in controls (C1 and C2) and NM fibroblasts (P1 and P3) by using the Seahorse analyzer and normalized to 15,000 cells as described in the Material and Methods. (**A**) Corresponding with P1; (**B**) corresponding with P3. Data represent the mean ± SD of three separate experiments. *** *p* < 0.001 between NM and controls cells; ^aa^ *p* < 0.01, ^aaa^ *p* < 0.001 between untreated (-) and treated NM cells.

We further examined the effect of LA and LCAR supplementation on mitochondrial function in NM fibroblasts using the bioenergetic profile provided by the Seahorse analyzer. Thus, mitochondrial bioenergetic parameters were examined in control and NM fibroblasts P1 (Figure 12A) and P3 (Figure 12B) treated and untreated with LA and LCAR. Supplementation with LA and LCAR individually or in combination was able to correct the reduced basal, maximal, and spare respiration, as well as mitochondrial ATP production in both ACTA1 (P1) and NEB (P3) mutant fibroblasts.

**Figure 13:**
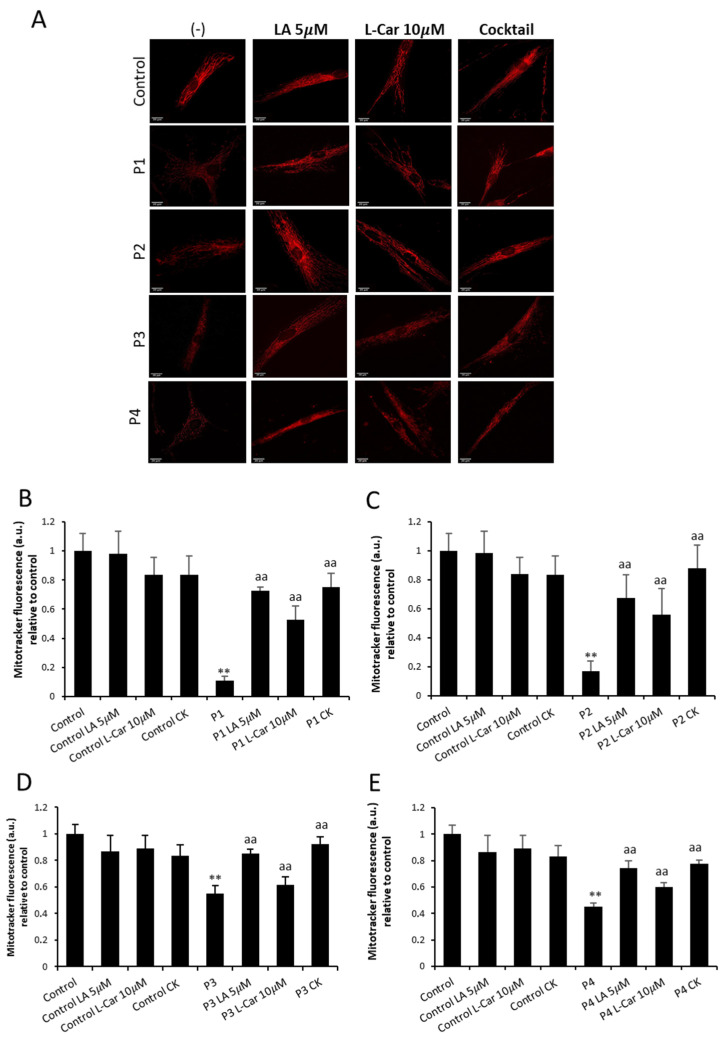
Effect of LA and LCAR on mitochondrial polarization and network in control and NM cells. Control (C1) and NM fibroblasts (P1, P2, P3, and P4) were treated (+) with 5 μM LA and 10 μM LCAR individually or in combination (Cocktail) for 7 days. (**A**) Representative images of untreated and treated control (C1) and NM fibroblasts (P1 and P2) stained with MitoTracker^TM^ Red CMXRos, an in vivo mitochondrial membrane-potential-dependent probe. Images were taken using a 40× lens and processed by ImageJ software. Scale bar = 20 µm. (**B**–**E**) Fluorescence quantification of MitoTracker signal. Data represent the mean ± SD of three separate experiments (at least 100 cells for each condition and experiment were analyzed). ** *p* < 0.01 between NM and controls cells; ^aa^ *p* < 0.01 between untreated (-) and treated NM cells. a.u.: arbitrary units.

Improvement of mitochondrial bioenergetics in mutant cells under LA and LCAR supplementation was also accompanied by a significant restoration of mitochondrial membrane potential and mitochondrial network morphology (Figure 13A). Furthermore, supplementation also restored the reduction in the percentages of tubular mitochondria found in NM cells compared to control cells (Supplementary Figure S7).

**Figure 14:**
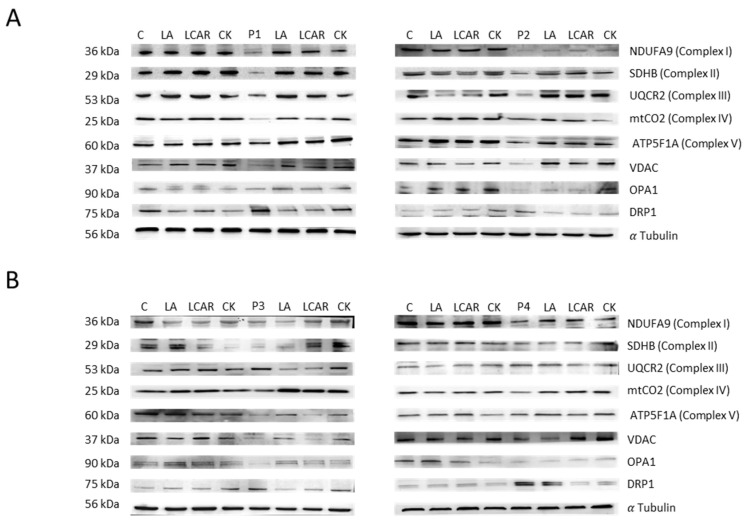
Effect of LA and LCAR on mitochondrial protein expression levels in control and NM cells. Control (C1) and NM fibroblasts (P1, P2, P3, and P4) were treated with 5 µM LA and 10 µM LCAR individually or in combination (Cocktail, CK) for 7 days. (**A**) Cellular extracts from control and NM patient cell lines P1 and P2 were subjected to immunoblotting analysis. (**B**) Cellular extracts from control and NM patient cell lines P3 and P4 were subjected to immunoblotting analysis. An SDS polyacrylamide gel was used to separate protein extracts (50 μg), then the samples were immunostained using antibodies against NDUFA9, SDHB, UQCR2, Mt-CO2, ATP5F1A, VDAC, OPA1, and DRP1. α-Tubulin was used as a loading control. (Densitometry of the Western blotting Supplementary Figures S8–S11).

In addition, LA and LCAR treatment notably increased mitochondrial protein expression levels (Figure 14 and Supplementary Figures S8–S11) and significantly reduced mitochondrial ROS production in NM cells (Supplementary Figure S12), thus confirming the improvement of mitochondrial function.

### 3.8. Supplementation with LA and LCAR Corrects RhoA/ROCK Pathway Overactivation

**Figure 15:**
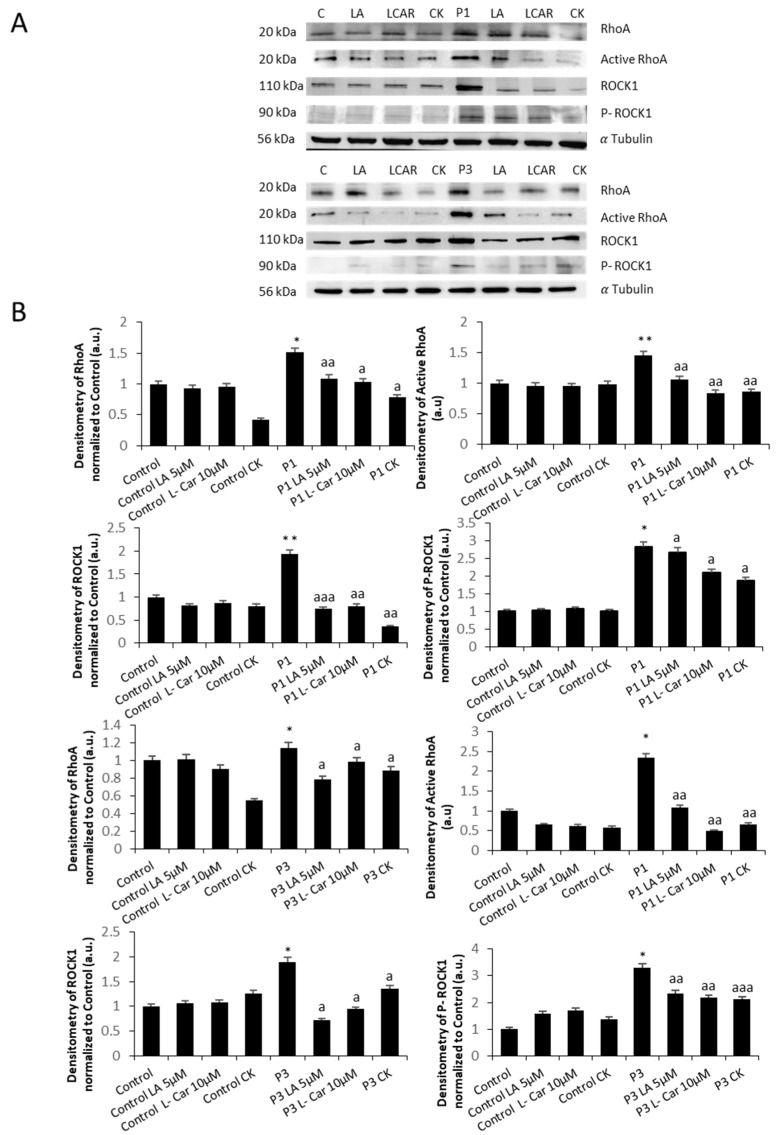
Effect of LA and LCAR on RhoA/ROCK pathway activation in control and NM cells. Control (C1) and NM fibroblasts (P1 and P2) were treated with 5 µM LA and 10 µM LCAR individually or in combination for 7 days. (**A**) Cellular extracts from control and NM patient cell lines P1 and P2 were subjected to immunoblotting analysis. An SDS polyacrylamide gel was used to separate protein extracts (50 μg), then the samples were immunostained using antibodies against RhoA, active RhoA purified by Active Rho Detection Kit as described in the Material and Methods, ROCK1, phospho-ROCK1, and α-tubulin, which was used as a loading control. (**B**) Densitometry of the Western blotting. For controls cells (C1 and C2), data are the mean ± SD of the two control cell lines. Data represent the mean ± SD of three separate experiments. * *p* < 0.05, ** *p* < 0.01 between treated and untreated (-) cells. ^a^ *p* < 0.05, ^aa^ *p* < 0.01, ^aaa^ *p* < 0.001 between untreated (-) and treated NM cells. a.u.: arbitrary units.

Next, to assess the effect of LA and LCAR on RhoA/ROCK pathway NM fibroblasts, we determined the expression levels of total RhoA, active RhoA, and downstream proteins (ROCK1 and pROCK1) in P1 and P3 NM fibroblasts (Figure 15A,B).

Results showed that LA and LCAR individually or in combination reduced the activation of RhoA and key essential downstream proteins such as pROCK1 both in ACTA1 (P1) and NEB (P3) fibroblasts.

## 4. Discussion

Nemaline myopathy (NM) encompasses a large spectrum of rare genetic myopathies characterized by hypotonia, weakness, and depressed or absent deep tendon reflexes. Histology examination of muscle biopsy typically shows the presence of nemaline rods. In this work, we explored the pathophysiological alterations in NM using patient-derived fibroblasts carrying *ACTA1* and *NEB* mutations. Mutant fibroblasts manifest alterations in actin filament polymerization associated with mitochondrial dysfunction. In addition, we identified two compounds, linoleic acid (LA) and L-carnitine (LCAR), that restored actin polymerization and corrected bioenergetics deficiency in both *ACTA1* and *NEB* mutant fibroblasts. Actin is a highly conserved and extensively distributed protein which is involved in many different biological processes and is essential to the cytoskeleton. Actin is involved in cell migration, cell division, and organelle transport in addition to giving the cell structural support [34,35]. Actin’s role was first described in muscle contraction in 1942 by A. Szent-Györgyi [36]. Actin is found in two different forms: G-actin and F-actin. Specifically, G-actin is the globular monomeric actin that polymerizes to form filamentous F-actin [37].

Specifically, higher mammals express six different isoforms of actin [38]. There are groups of structurally related genes with highly homologous nucleotide sequences that share a common precursor code for different isoforms of actin [39]. Actin isoforms differ by four amino acid residues localized at positions 1, 2, 3, and 9 of the N-terminus [38]. Six human actin genes, α-skeletal (*ACTA1*), α-cardiac (*ACTC1*), α-smooth muscle (*ACTA2*), γ-smooth muscle (*ACTG2*), β-cytoplasmic (*ACTB*), γ-cytoplasmic (*ACTG1*)—are localized on the different chromosomes [40]. The seventh actin isoform, β-actin-like protein 2 (ACTBL2), has been recently identified. It is a member of the non-muscle actin class, along with γ- and β-cytoplasmic actins, but its expression is extremely low [41]. Cytoplasmic actins are expressed in mammalian cells in various proportions [42]. In particular, cytoplasmic β- and γ-actins are the isoforms mainly expressed in fibroblasts. However, it is well known that actin isoforms can act redundantly [43,44,45]. For example, Tondeleir et al. demonstrated that impaired migration of mouse embryonic fibroblasts caused by ablation of the β-actin isoform can be restored by an upregulation of α- and γ-actin isoforms, suggesting that these isoforms can work redundantly and thus compensate for each other’s loss [45]. Moradi et al. also showed how depletion of each actin isoform (α-actin, β-actin, or γ-actin) might induce a compensatory upregulation of other isoforms in motor neurons [43].

Specifically, in our study we have shown that ACTA1 mutant fibroblasts (P1, P2) had reduced levels of both gene expression of the ACTA1 gene and α-actin protein expression levels compared to control cells (Figure 1, Figure 3, and Figure 4A). However, both ACTA1 and NEB fibroblasts showed defects in actin filament polymerization (Figure 5) and increased protein expression levels of the β-actin isoform in comparison with control cells (Figure 3C,D). Therefore, as has been previously described, a compensatory effect of actin isoforms could be occurring in the case of our mutant cells. The elevated protein expression levels of β-actin shown by mutant cells compared to control could be acting as a compensatory mechanism to correct the defects in actin filament polymerization presented by ACTA1 and NEB mutant fibroblasts.

On the other hand, nebulin (NEB) is a giant filamentous protein with a size from 600 to 900 kDa that is an essential part of the thin filament in skeletal muscle. Many of its functions are still mostly unknown due to its size and the difficulty in obtaining nebulin in its native state from muscle [46]. However, nebulin is crucial in the regulation of actin filament length, the interaction between actin and myosin, myofilament calcium sensitivity, and, consequently, in the control of muscle contraction. Particularly, in our study we have shown that NEB mutant fibroblasts (P3, P4) had reduced levels of both gene expression of the NEB gene and nebulin protein expression levels compared to control cells (Figure 2 and Figure 4B). Curiously, NEB transcript levels in ACTA1 mutant cells (P1, P2) were significantly higher than the expression levels shown by control cells (Figure 4B). Numerous previously published studies have demonstrated that there is a strict regulation between actin and nebulin. Specifically, nebulin expression is modified by α-actin mutations, nebulin may appear altered as a result of a primary defect in α-actin that severely disrupts the sarcomeric thin filament, and, additionally, the binding of nebulin to F-actin may also be affected by nebulin mutations [26,47,48]. Therefore, our results suggest that NEB overexpression in ACTA1 mutant cells (P1, P2) may be acting as a compensation mechanism for defects in ACTA1, due to the important role nebulin plays in regulating the length of and stabilizing actin filaments.

In our study, we have demonstrated for the first time that both actin alpha 1 (ACTA1) and nebulin (NEB) are expressed in dermal fibroblasts, allowing the investigation of pathological alterations induced by mutant proteins in an easy-to-use cellular model. In addition, both proteins indeed participate in actin filament polymerization in dermal fibroblasts since both mutant proteins ACTA1 and NEB caused defects in actin polymerization in NM cells.

One interesting finding in our work is that NM-patient-derived fibroblasts exhibited alterations in mitochondrial network morphology associated with downregulation of the expression levels of mitochondrial proteins and deficient mitochondrial bioenergetics, suggesting a marked mitochondrial dysfunction. Mitochondria are organelles that undergo dynamic changes through fission (division into two or more independent organelles) and fusion (formation of a single structure) events, biogenesis, and mitophagy (clearance of damaged organelles) [49,50]. Referred to as mitochondrial quality control, these processes regulate the number, shape, and turnover of mitochondria, respectively [51]. In mammalian cells, dynamin-related protein 1 (DRP1), mitofusin 1 (MFN1), mitofusin 2 (MFN2), and optic atrophy protein 1 (OPA1) regulate the adaptive alterations known as mitochondrial fusion and fission cycles [52]. It has been observed that mitochondrial fission is a prerequisite for mitophagy and contributes to mitochondrial apoptosis, whereas mitochondrial fusion enhances mitochondrial metabolism [53]. Mitophagy removes selectively old or dysfunctional mitochondria through sequestration and engulfment for the subsequent lysosomal degradation [54] and it is also enhanced by different stresses, such as oxidative damage, hypoxia, mitochondrial depolarization, or mitochondrial DNA damage [55,56]. Accumulating evidence reveals that mitophagy is necessary to preserve skeletal muscle plasticity by controlling mitochondrial biogenesis turnover, and mitochondrial proteostasis [57,58], in order to increase mitochondrial activity and remodeling during early myogenic differentiation [59,60,61].

Is well known that actin polymerization is crucial for mitochondrial function. Specifically, mitochondrial fission is stimulated by actin polymerization from the endoplasmic reticulum via formin INF2, while other forms of mitochondrial fission are dependent on actin polymerization that is mediated by the Arp2/3 complex [62]. Actin can both promote and impede mitochondrial motility. More investigation is necessary to understand the precise mechanisms, as the parameters governing actin’s mitochondrial localization during these processes remain poorly known. Investigations are also being conducted on the entry of actin nucleators and other actin-binding proteins into mitochondria during actin-dependent activities [62].

Cytoskeletal components, particularly microtubules and F-actin, work collaboratively to control the morphology, mitophagy, and fission/fusion mitochondrial processes in response to extracellular stimuli or stressors [63]. Mitochondrial motility is also dependent on the coordinated action of cytoskeletal elements, especially microtubules and F-actin, which distribute and anchor the organelles to the proper locations within the cell [64]. Specifically, it has been proposed that changes to the cytoskeleton may affect mitochondria, leading to functional modifications in the organelle. The primary role of mitochondria in cells is energy production, which is closely correlated with the regulation of their morphology, organization, and distribution by the cytoskeleton [65]. Thus, cytoskeletal abnormalities of actin polymerization in NM can subsequently lead to impairments in mitochondrial respiration, thereby accelerating disease progression.

The process of mitochondrial fission involves the formation of ring-shaped DRP1 oligomers on the outer membrane of the mitochondria, which are then constricted by hydrolysis [66]. In order to facilitate the subsequent recruitment of dynamin-2, which coordinates membrane scission, DRP1 rings tighten and constrict mitochondria [67]. Nevertheless, in order to reduce their cross-sectional diameter, mitochondria must first go through a pre-constriction step because they are frequently thicker than DRP1 rings. Endoplasmic reticulum (ER) tubules are wrapped and tightened around mitochondria to produce this pre-constriction. Growing evidence points to actin polymerization as the primary force behind the ER’s bending around the mitochondria [68,69].

In addition, F-actin at Mito/ER contacts may prime DRP1 to form functional oligomers on the mitochondrial outer membrane [70]. DRP1 activity is greatly increased in the presence of actin filaments, according to in vitro GTPase assays [70]. These findings suggest that filamentous actin is an important regulator of inner and outer mitochondrial membrane fission.

Moreover, the actin cytoskeleton controls the movement of mitochondria in simple eukaryotes like budding yeast [71] and is necessary for the accurate inheritance of mitochondria during cytokinesis [72]. Microtubules in metazoans are responsible for coordinating long-distance mitochondrial movement, but the actin cytoskeleton is also involved in regulating mitochondrial distribution, coordinating anchoring, and coordinating short-distance mitochondrial motility [63].

Furthermore, the expression and maintenance of mtDNA are significantly influenced by the actin cytoskeleton [73]. The mammalian mitochondrial DNA genome (mtDNA) contains 37 genes organized in compact DNA:protein complexes known as nucleoids [74,75], whose expression requires a precise coordination with the nuclear genome [76]. In yeast, the ER–mitochondria encounter structure (ERMES) complex controls the stability and arrangement of mtDNA in nucleoids in an actin-dependent way [77]; in mammals, the distribution, division, and active transport of mitochondrial nucleoids by microtubules are regulated by mitochondria endoplasmic reticulum contact sites (MERCs), which are spatially linked to these nucleoids [78]. Further, recent super-resolution microscopy-based studies probed the existence of β-actin-containing structures within the mitochondrial matrix [79].

Additionally, altered mtDNA mass and nucleoid organization, as well as stress induced by a reduction in mitochondrial membrane potential (ΔΨ), were more common in β-actin-deficient human cells, suggesting a regulatory role for β-actin in mtDNA transcription and quality control [80]. In addition to actin, myosin II has also been associated with isolated mitochondrial nucleoids, and its silencing results in mtDNA alterations [73]. These data support the role of actin and actin-binding proteins in mitochondrial nucleoid segregation and mtDNA transcription and preservation, likely through formation of a “mitoskeleton” network supporting mtDNA inheritance.

Additionally, the complete activation of metabolic pathways that subsequently control mitochondrial function depends on actin filaments. For example, direct binding of glycolytic enzymes like glyceraldehyde phosphate dehydrogenase or aldolase to F-actin can activate them [81]. Interestingly, actin regulates cytochrome c retention between respiratory chain complexes III and IV in brain mitochondria via direct association with both complexes, and inhibition of actin polymerization with cytochalasin b increased mitochondrial respiration via increased complex IV activity [82]. Recently, proteomic analysis using NEB [83] and ACTA1 [84] mouse models showed alterations in several cellular processes and functions, including mitochondrial dysfunction and changes in energetic metabolism and stress-related pathways. Abnormal mitochondrial distribution, reduced mitochondrial respiratory function, increased mitochondrial membrane potential, and abnormally low ATP content were all revealed by structural and functional studies.

In our work, we have also identified two compounds, LA and LCAR, that by improving mitochondrial bioenergetics were able to restore actin polymerization.

LA is a carboxylic acid composed of 18 carbon atoms and three cis double bonds (18:2ω6). It is an essential fatty acid required by the human body and must therefore be consumed through the diet [85,86]. Pharmacological studies have shown that LA has a wide range of pharmacological effects such as anti-metabolic syndrome, anti-inflammatory, anti-cancer, antioxidant, neuroprotection, and the regulation of the intestinal flora [87,88,89]. LA is a major component of cardiolipin (CL), which is a specific inner-mitochondrial membrane phospholipid that is important for optimal mitochondrial function including respiration and energy production [90]. Furthermore, this phospholipid participates in morphology and stability of mitochondrial cristae, fission- and fusion-mediated mitochondrial quality control and dynamics, mitophagy, mitochondrial biogenesis and protein import, and multiple mitochondrial steps of the apoptotic process [90]. CL is particularly prone to peroxidation, an event that may affect many CL-dependent reactions and processes, due to its high content of unsaturated fatty acids and its location in the IMM near electron transport chain (ETC) complexes, the main sites of reactive oxygen species (ROS) production [91,92,93]. Furthermore, oxidized CL accumulation in the OMM serves as an important signaling platform during the apoptotic process, resulting in the opening of the mitochondrial permeability transition pore (mPTP) and the release of cytochrome c (cyt c) from mitochondria to the cytosol [94,95].

Specifically, CL and its LA content are known to be positively associated with cytochrome c oxidase (COX) activity [96], thereby making this lipid molecule an essential factor related to mitochondrial health and function. Structural studies have shown that CL is important for stabilization (i.e., the assembly of complex subunits) of mitochondrial oxidative phosphorylation (OXPHOS) complex I (CI), complex II (CII), complex III (CIII), and complex IV (CIV) [97,98,99,100]. On the other hand, a decrease in CL levels by low dietary intake of LA promotes the disassembly of these mitochondrial OXPHOS complexes [101]. In contrast, an LA-enriched diet increased mitochondrial CL and OXPHOS protein levels [102]. Additionally, it was recently discovered that cardiolipin is necessary for the respiratory chain to organize into supramolecular assemblies [103]. Furthermore, incubation of cells with different concentrations of LA led to a dose- and time-dependent increase in cardiolipin levels [104].

It is possible that supplementing with LA by raising cardiolipin levels will have a beneficial effect on the activity of many different mitochondrial proteins and enzymes, such as the complexes involved in oxidative phosphorylation (OXPHOS) and the electron transport chain (ETC), thereby enhancing mitochondrial dynamics and function. Furthermore, LA stimulates mitochondrial biogenesis signaling by the upregulation of PPARγ coactivator 1α (PGC-1α) in C2C12 cells [105].

In addition to its cardiolipin-dependent actions, LA also promotes an increase in fascin expression, an actin crosslinker globular protein that generates actin bundles built of parallel actin filaments, which mediate formation and stability of cellular protrusions including microspikes, stress fibers, membrane ruffles, and filopodia [106]. Accordingly, LA is found to promote phagocytic cup formation and membrane ruffling along with cytoskeletal reorganization in microglia [107]. Furthermore, LA has many effects on cytoskeleton proteins in general and actin filaments in particular. For instance, it has been observed that LA promotes cell migration by modulating the microtubule dynamics and actin cytoskeleton remodeling at the forefront of the cell by formation of lamellipodia [108]. Moreover, LA may activate the RhoA/ROCK pathway and, therefore, may facilitate actin polymerization. Thus, it has been shown that LA increased intercellular adhesion molecule 1 (ICAM-1) expression and phosphorylation of ROCK and myosin phosphatase target subunit 1 (MYPT-1), a distal signal of ROCK [109]. However, the direct effect of LA supplementation on the RhoA/ROCK pathway in NM cellular models needs further investigation.

On the other hand, LCAR is an available dietary supplement which is required for the translocation of fatty acids into the mitochondrial compartment for β-oxidation [110,111]. LCAR also has the potential to increase mitochondrial biogenesis by increasing the gene expression of various mitochondrial components and maintaining their function by supplying their respective substrates and protecting them from insults such as the accumulation of toxic products or reactive radicals [112,113]. Therefore, LCAR as a natural compound that can enhance cellular energy transduction may have therapeutical potential in NM. Studies in recent years have demonstrated the protective effects of LCAR treatment on mitochondrial functions [113]. Consistent with this hypothesis, a premature boy with a congenital form of nemaline myopathy due to mutation in the *ACTA1* gene showed decreased LCAR levels in the eighth week of life. After sufficient oral LCAR substitution he improved gradually [114]. Furthermore, LCAR ameliorates congenital myopathy in a tropomyosin 3 de novo mutation transgenic zebrafish [115]. In addition, LCAR significantly reduced statin-induced myopathy [116] and skeletal muscle atrophy in rats [117]. Moreover, LCAR improved exercise performance in human patients with mitochondrial myopathy [118] and may prevent age-associated muscle protein degradation and regulate mitochondrial homeostasis [119].

During cycles of actin assembly and disassembly, actin monomers polymerize into filaments in an adenosine-triphosphate (ATP)-bound state, and the polymerization of actin is followed by the irreversible hydrolysis of ATP to adenosine diphosphate (ADP) and phosphate (6). Thus, in eukaryotic cells, the actin cytoskeleton’s turnover between its monomeric and filamentous forms is an ATP-dependent process and a significant energy drain. For this reason, it is not surprising that lower ATP levels slow actin polymerization since ATP is essential for actin polymerization. Reduced ATP levels most likely cause an abundance of ADP-G-actin versus ATP-G-actin. The lower actin polymerization rate observed in the ATP-deficient cells may be explained by the fact that ADP-G-actin polymerizes at a substantially slower rate than ATP-G-actin [120,121]. Actin filament assembly and dynamic behavior are mediated in large part by binding with ATP and ATP hydrolysis.

In addition, RhoA activity, which regulates actin polymerization, decreased in parallel with the concentration of ATP and GTP during depletion by mitochondrial inhibitors and recovered rapidly when cells were returned to normal culture conditions [122], suggesting that ATP and GTP levels modulate RhoA activation.

In this regard, mitochondrial dysfunction in NM cells may cause ATP deficiency and aggravate the impairment of actin polymerization. LCAR and/or LA supplementation may boost mitochondrial ATP formation and consequently may facilitate actin filament formation by increasing actin monomers bound to ATP and RhoA activation by increasing GTP levels.

## 5. Conclusions

In conclusion, our findings demonstrate that fibroblasts derived from NM patients are useful cellular models to achieve a better understanding of the disease and to evaluate the effectiveness of pharmacological compounds. Furthermore, we confirm the close relationship between actin cytoskeleton and mitochondrial function. Supplementation with LA or/and LCAR was able to restore actin polymerization and correct mitochondrial dysfunction in NM cells. Further studies are required to assess the benefit of both compounds in sarcomere organization and skeletal muscle contraction. Patient-derived cellular models may complement *ACTA1* and *NEB* mouse and zebrafish models and enable the evaluation of genomic or pharmacological therapies.

## Data Availability

Data and material are available under request.

## Supplementary Materials

The following supporting information can be downloaded at: https://www.mdpi.com/article/10.3390/antiox12122023/s1, Figure S1: Expression level of cytoskeletal components of NM cells; Figure S2: RhoA/ROCK pathway inhibition aggravated the state of actin polymerization in control and NM cells; Figure S3: Dose–response curve of the effect of LA and LCAR on actin polymerization in patient P1 cells; Figure S4: Dose–response curve of the effect of LA and LCAR on actin polymerization in patient P2 cells; Figure S5: Dose–response curve of the effect of LA and LCAR on actin polymerization in patient P3 cells; Figure S6: Dose–response curve of the effect of LA and LCAR on actin polymerization in patient P4 cells; Figure S7: Quantification of tubular and rounded percentages of mitochondria in control and NM fibroblasts; Figure S8: Densitometry of the Western blotting of patient P1 corresponding to Figure 13A; Figure S9: Densitometry of the Western blotting of patient P2 corresponding to Figure 13A; Figure S10: Densitometry of the Western blotting of patient P3 corresponding to Figure 13B; Figure S11: Densitometry of the Western blotting of patient P4 corresponding to Figure 13B; Figure S12: Measurement of mitochondrial reactive oxygen species (ROS) generation in treated and untreated control and NM cells (P1 and P3).
