# Supplementary material for: Actin Polymerization Defects Induce Mitochondrial Dysfunction in Cellular Models of Nemaline Myopathies
Source: Antioxidants (Basel). 2023 Nov 21;12(12):2023. doi: 10.3390/antiox12122023 (PMC10740811; doi:10.3390/antiox12122023)
Supplement: Supplementary file 1 [file antioxidants-12-02023-s001.zip › antioxidants-2707573-supplementary.pdf]

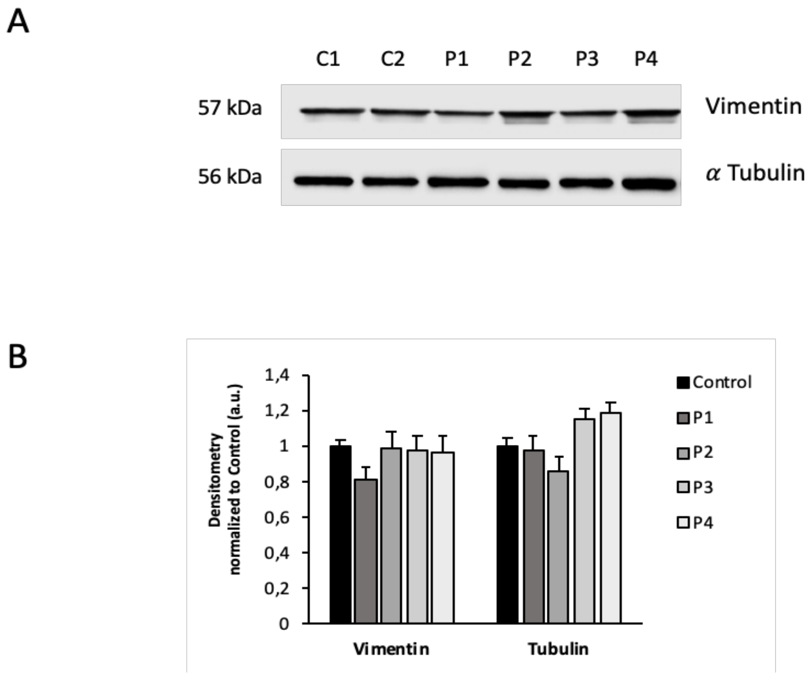

Supplementary Figure S 1. Expression level of cytoskeletal components of NM cells. (**A**) Immunoblotting analysis of cellular extracts from controls (C1 and C2) and NM patient cell lines P1, P2, P3 and P4. Protein extracts (50 µg) were separated on a SDS polyacrylamide gel and immunostained with antibodies against Vimentin and α Tubulin, which was used as a loading control. (**B**) Densitometry of the Western blotting. For controls cells (C1 and C2), data are the mean±SD of the two control cell lines. Data represent the mean±SD of three separate experiments. *p<0.05 between NM and controls cells. A.U., arbitrary units.

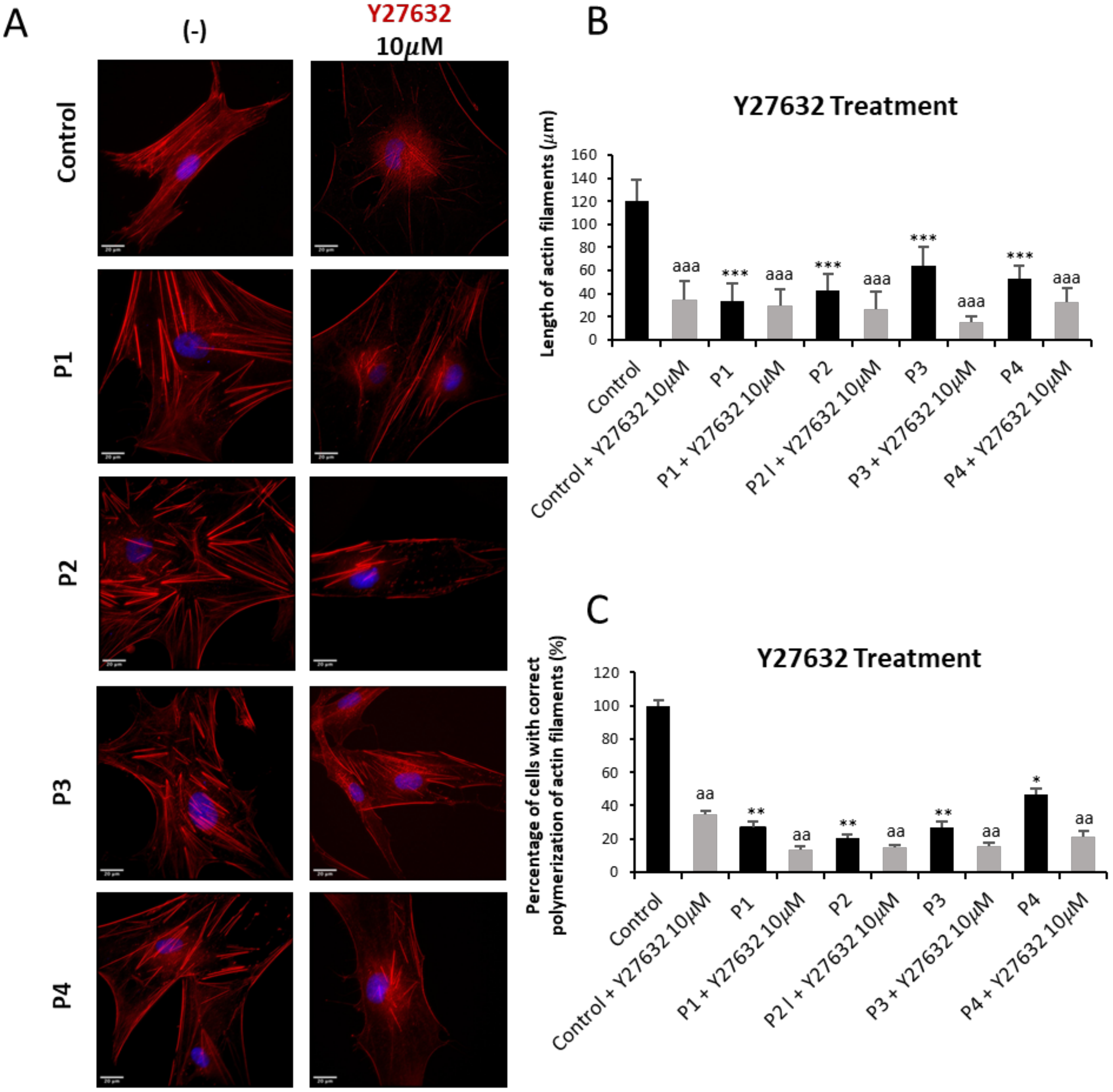

Supplementary Figure S2. RhoA/ROCK pathway inhibition aggravated the state of actin polymerization in control and NM cells. (**A**)Control (C1) and NM cells (P1, P2, P3 and P4) were treated for 24 hours in the presence or absence of 10 μM Y-27632. Control and NM cells, P1 and P2 (mutation in *ACTA1*) and, P3 and P4 (mutation in *NEB*) were stained with phalloidin-rhodamine, following Materials and Methods. Images were taken using the 40x lens and processed by the ImageJ software. (**B**) Measurements of the length of actin filaments (μm). The length of the actin filaments was measured in triplicate with the ImageJ software in 30 images (**C**) Quantification of the percentage of cells with correct actins polymerization. *p< 0.05, **p<0.01, ***p<0.001 between controls cells and NM cells; aa p<0.01, aaa p<0.001 between untreated and treated NM cells.

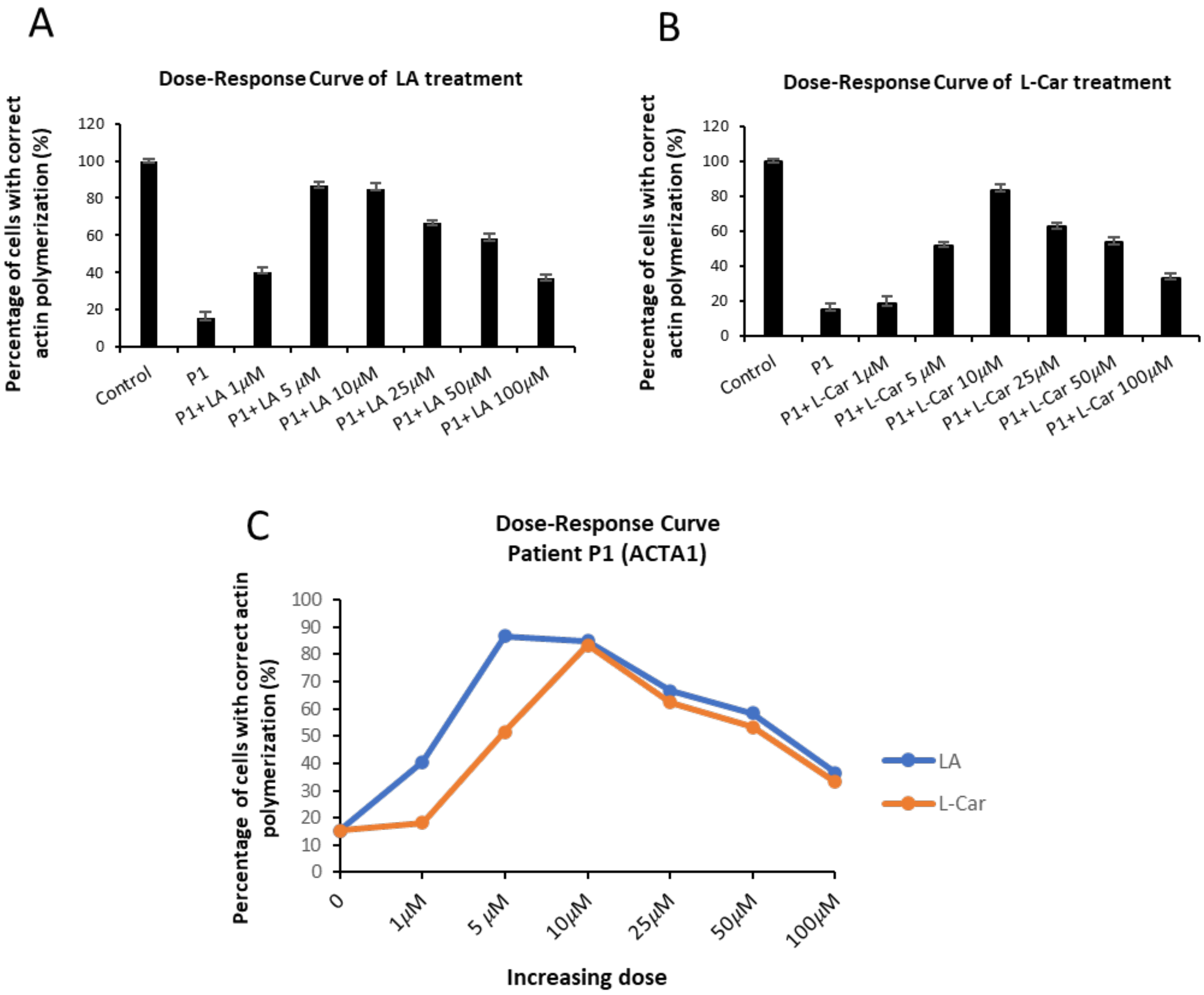

Supplementary Figure S 3. Dose-response curve of the effect of LA and LCAR on actin polymerization in patient P1 cells. (**A**) and (**B**) NM cells were treated with increasing concentrations of LA and LCAR (1, 5,10, 25, 50 and 100 μM) and determining the percentage of cells with correct actin polymerization. (**C**) Dose-response curve of patient P1 of the effect of both treatments.

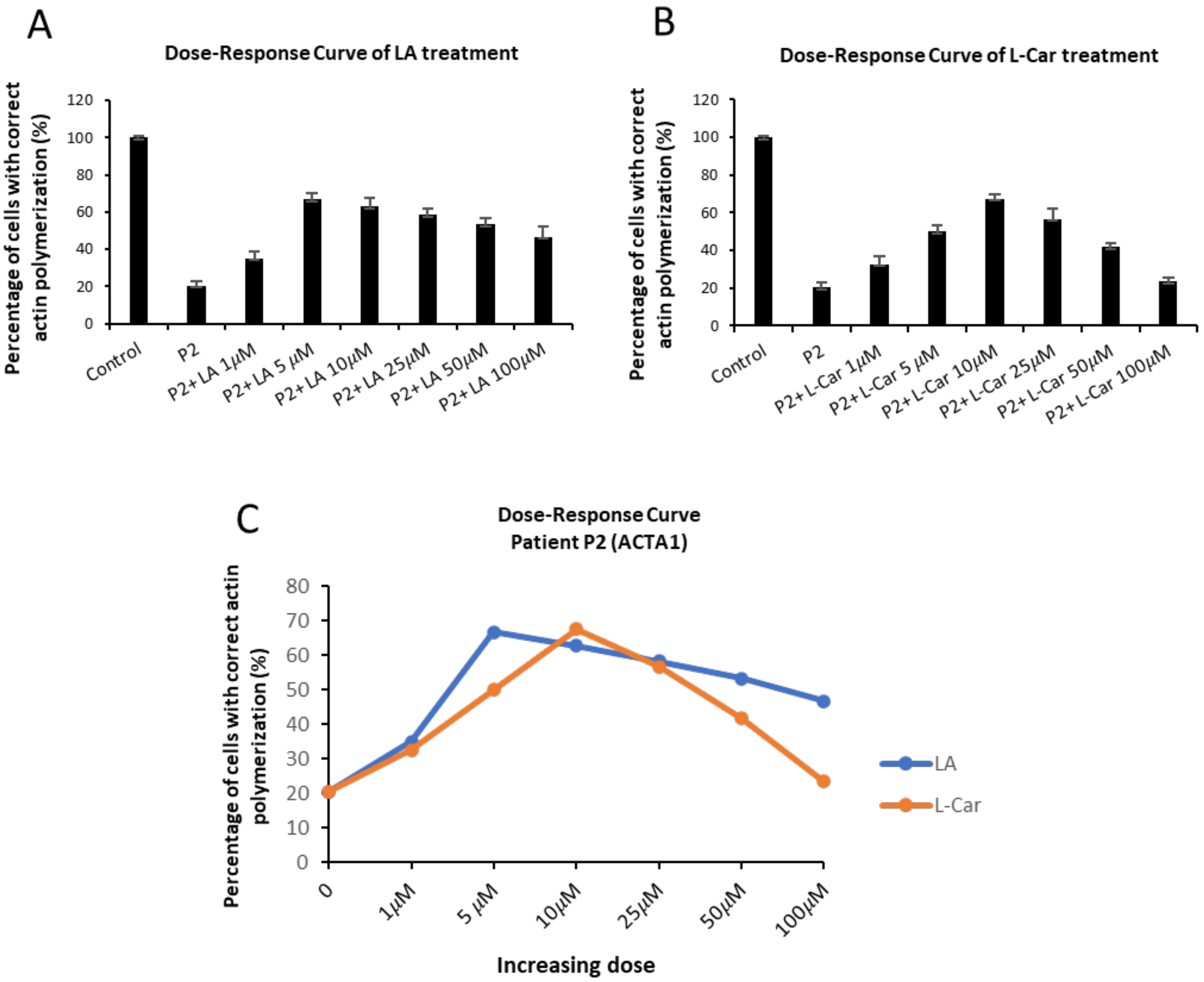

Supplementary Figure S4. Dose-response curve of the effect of LA and LCAR on actin polymerization in patient P2 cells. (**A**) and (**B**) NM cells were treated with increasing concentrations of LA and LCAR (1, 5,10, 25, 50 and 100 µM) and determining the percentage of cells with correct actin polymerization. (**C**) Dose-response curve of patient P2 of the effect of both treatments.

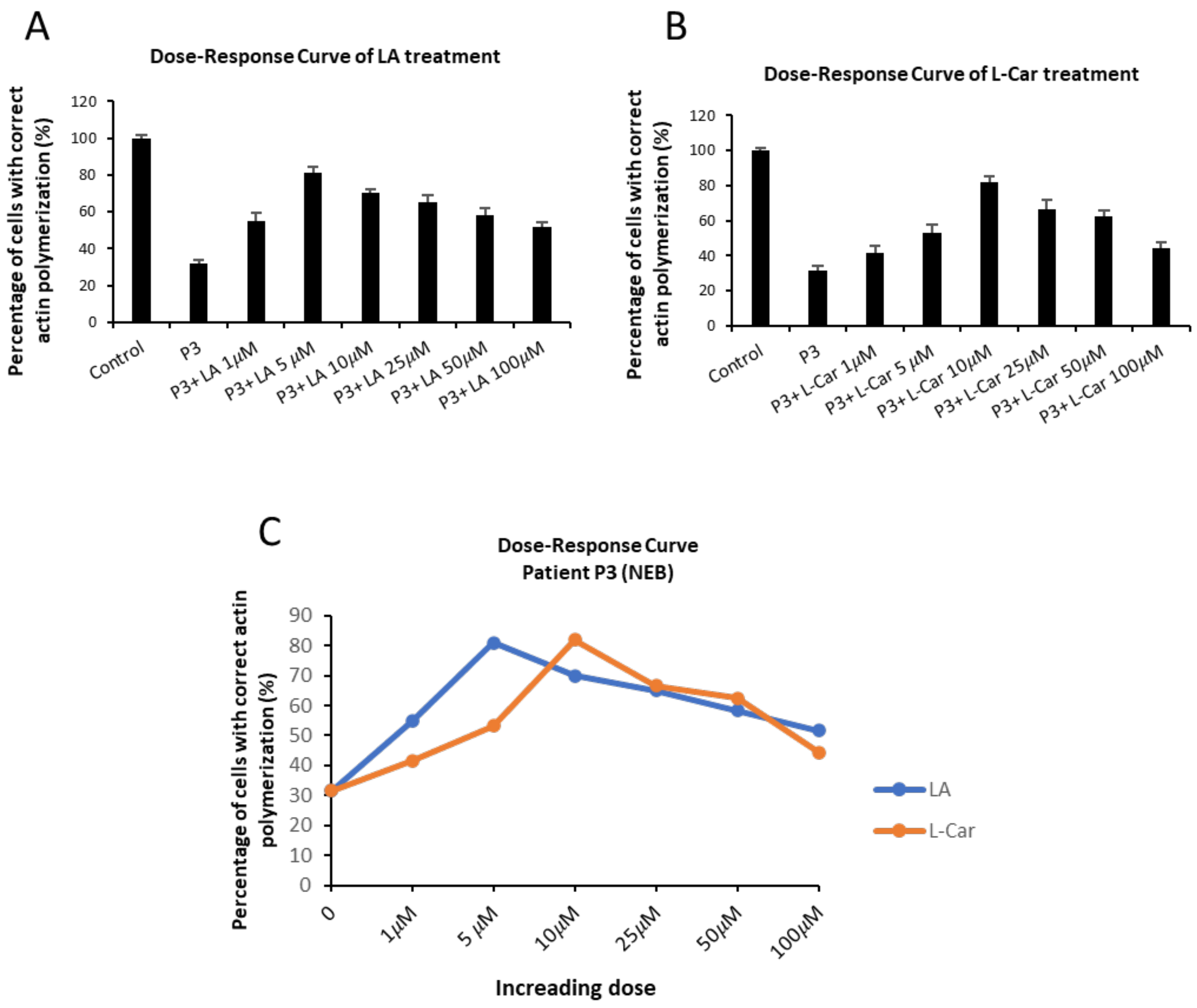

Supplementary Figure S 5. Dose-response curve of the effect of LA and LCAR on actin polymerization in patient P3 cells. (**A**) and (**B**) NM cells were treated with increasing concentrations of LA and LCAR (1, 5,10, 25, 50 and 100 μM) and determining the percentage of cells with correct actin polymerization. (**C**) Dose-response curve of patient P3 of the effect of both treatments.

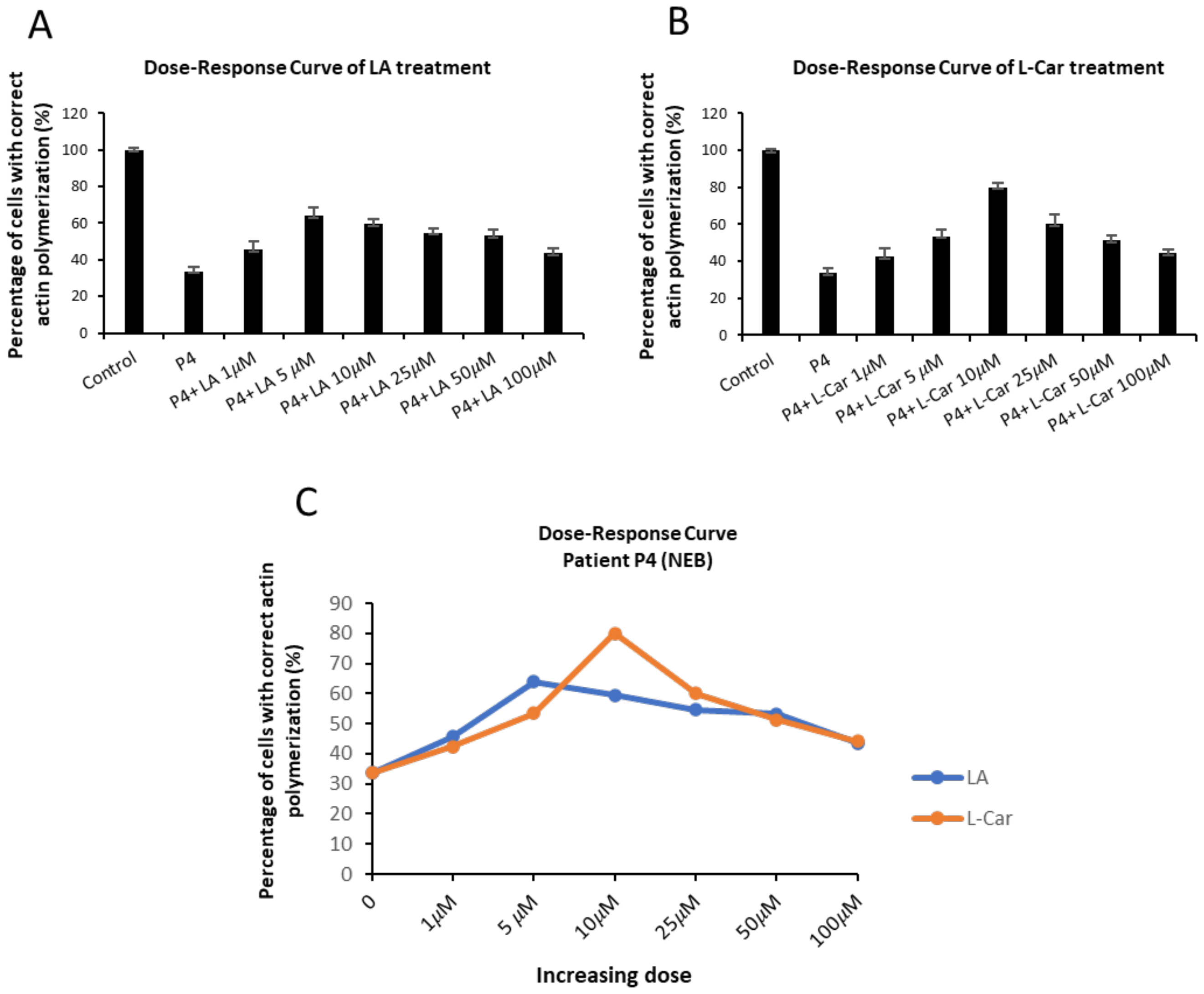

Supplementary Figure S 6. Dose-response curve of the effect of LA and LCAR on actin polymerization in patient P4 cells. (**A**) and (**B**) NM cells were treated with increasing concentrations of LA and LCAR (1, 5,10, 25, 50 and 100 µM) and determining the percentage of cells with correct actin polymerization. (**C**) Dose-response curve of patient P4 of the effect of both treatments.

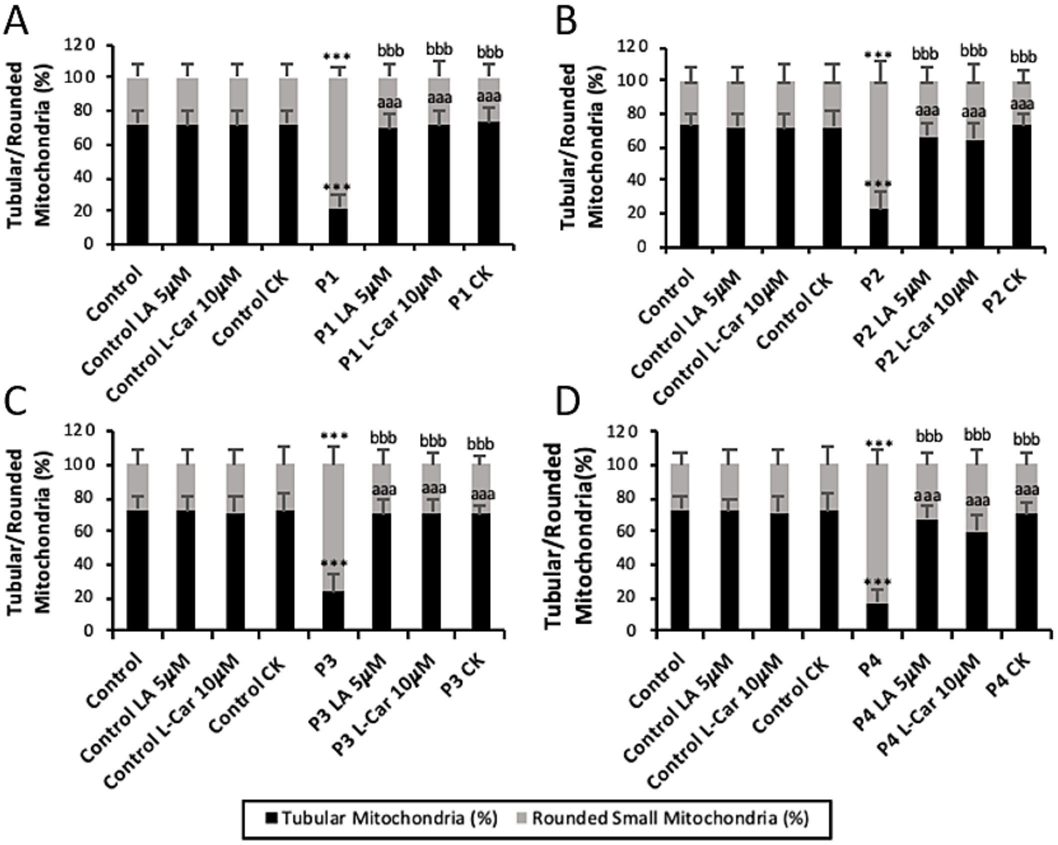

Supplementary Figure S 7. Quantification of tubular and rounded percentage of mitochondria in control and NM fibroblasts. Data represent the mean ± SD of three separate experiments (at least 100 cells for each condition and experiment were analyzed). ***$p<0.001$ between NM cells and controls; a $p<0.05$, aaa $p<0.001$ between untreated and treated NM cells of tubular mitochondria percentage; bbb $p<0.001$ between untreated and treated NM cells of rounded mitochondria percentage. A. U.: arbitrary units.

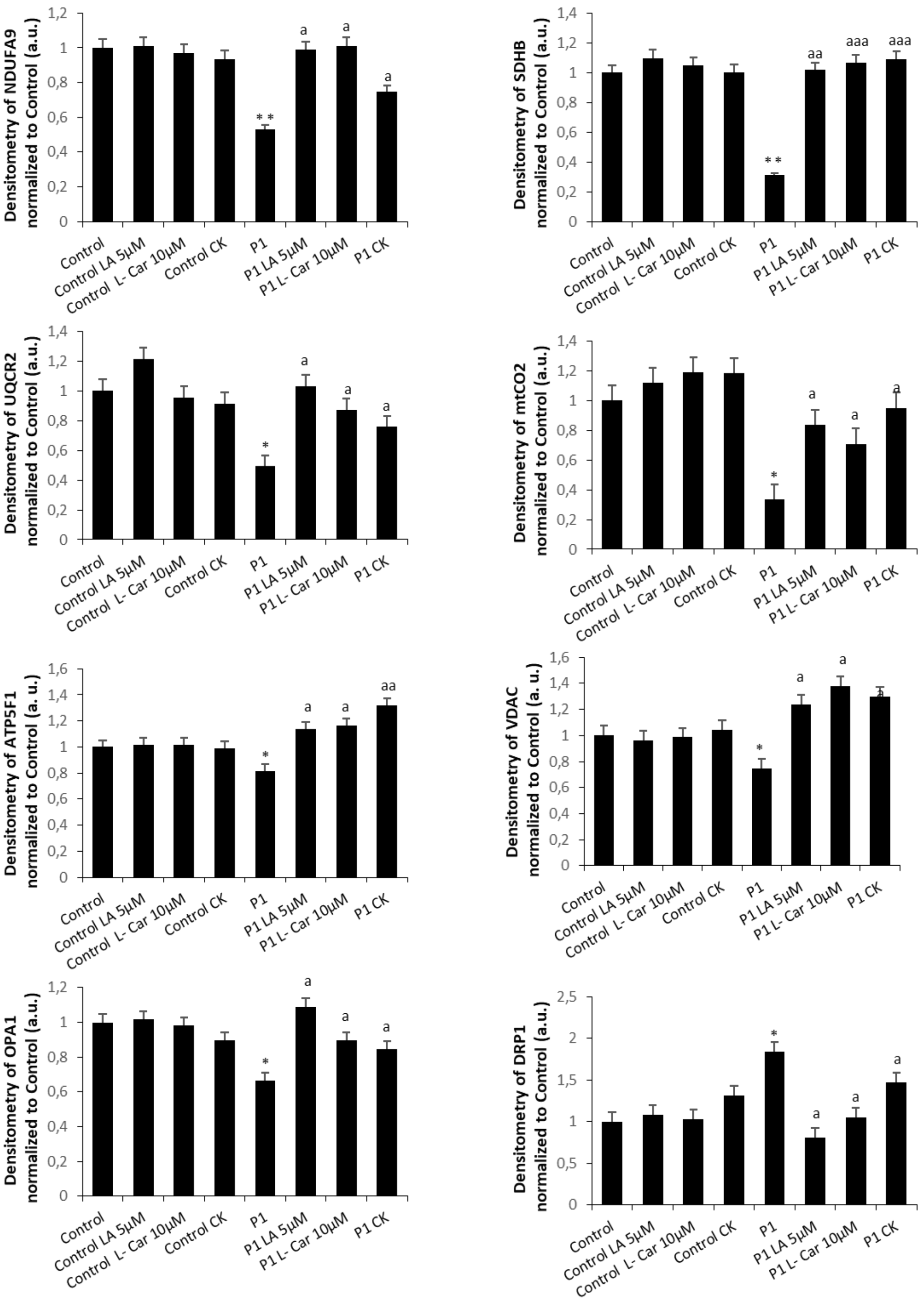

Supplementary Figure S8. Densitometry of the Western blotting of patient P1 corresponding to figure 13A. For controls cells (C1 and C2), data are the mean±SD of the two control cell lines. Data represent the mean±SD of three separate experiments. *p<0.05, **p<0.01, between treated and untreated (-) cells. $^{a}p<0.05$ $^{aa}p<0.01$ $^{aaa}p<0.001$ between untreated (-) and treated NM cells. A.U., arbitrary units.

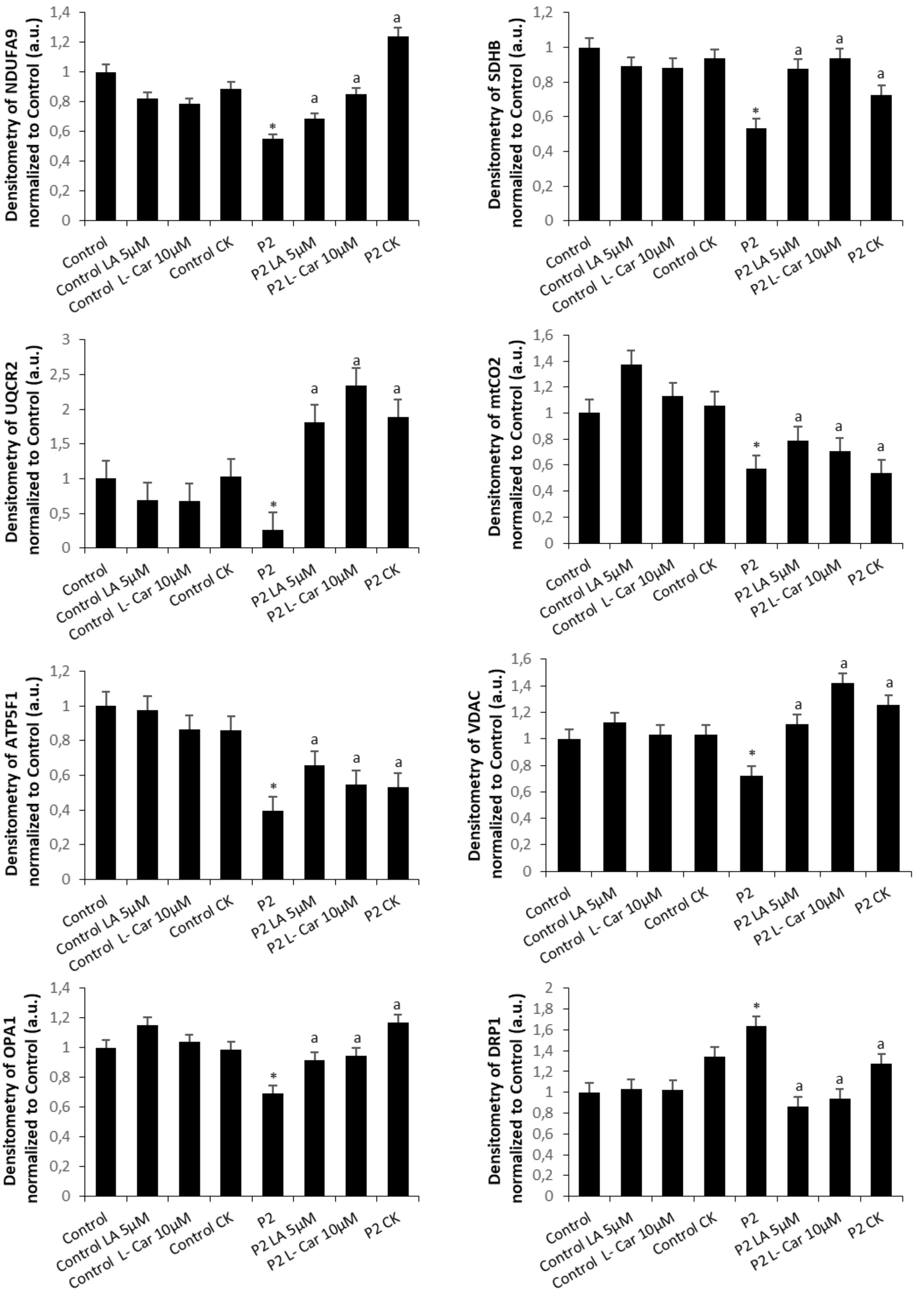

Supplementary Figure S9. Densitometry of the Western blotting of patient P2 corresponding to figure 13A. For controls cells (C1 and C2), data are the mean±SD of the two control cell lines. Data represent the mean±SD of three separate experiments. $*p<0.05$, $**p<0.01$, between treated and untreated (-) cells. $^{a}p<0.05$ between untreated (-) and treated NM cells. A.U., arbitrary units.

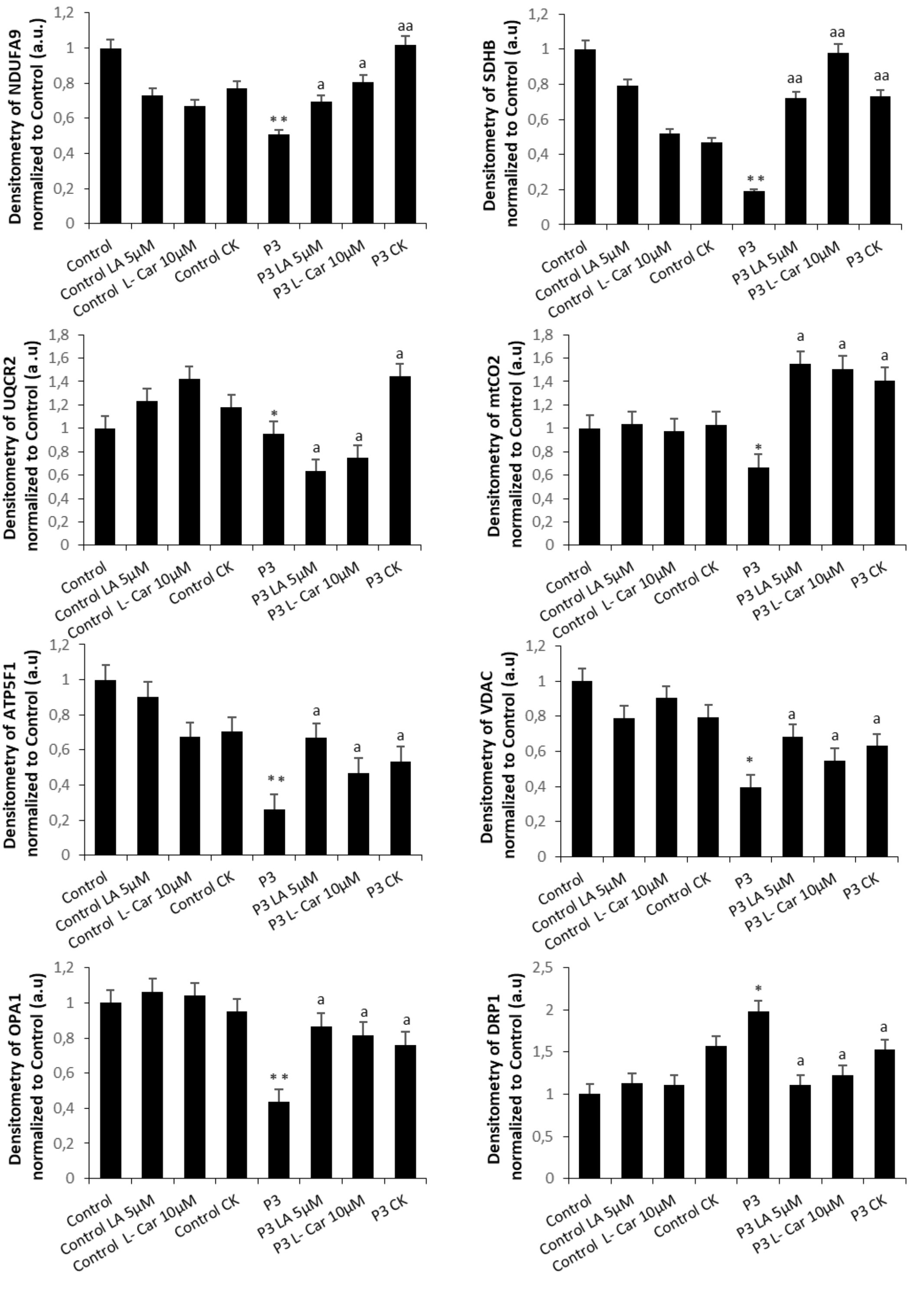

Supplementary Figure S 10. Densitometry of the Western blotting of patient P3 corresponding to figure 13B. For controls cells (C1 and C2), data are the mean±SD of the two control cell lines. Data represent the mean±SD of three separate experiments. *p<0.05, **p<0.01, between treated and untreated (-) cells. [a]p<0.05 [aa]p<0.01 between untreated (-) and treated NM cells. A.U., arbitrary units.

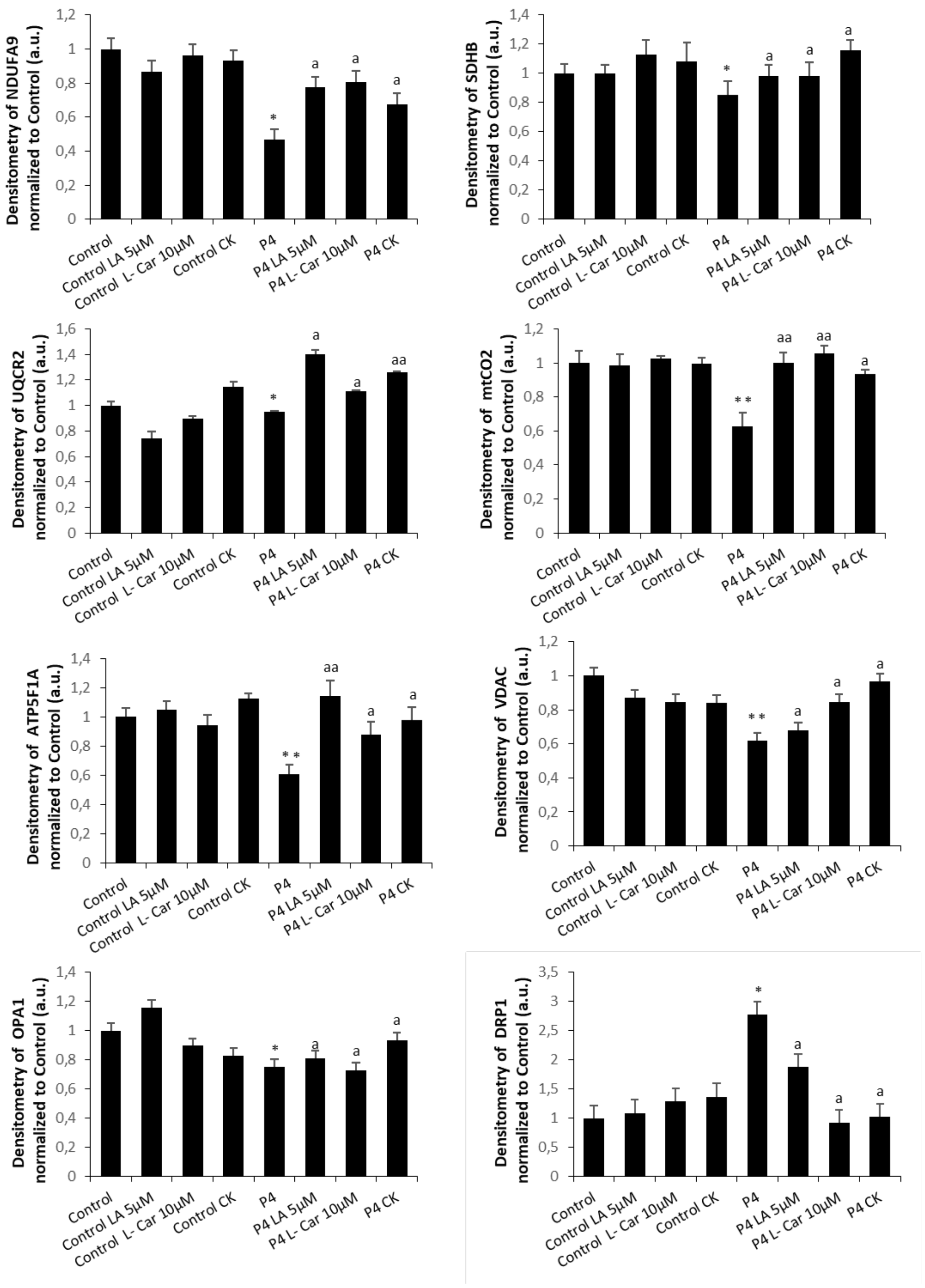

Supplementary Figure S 11. Densitometry of the Western blotting of patient P4 corresponding to figure 13B. For controls cells (C1 and C2), data are the mean±SD of the two control cell lines. Data represent the mean±SD of three separate experiments. *p<0.05, **p<0.01, between treated and untreated (-) cells. ^{a}p<0.05 ^{aa}p<0.01 between untreated (-) and treated NM cells. A.U., arbitrary units.

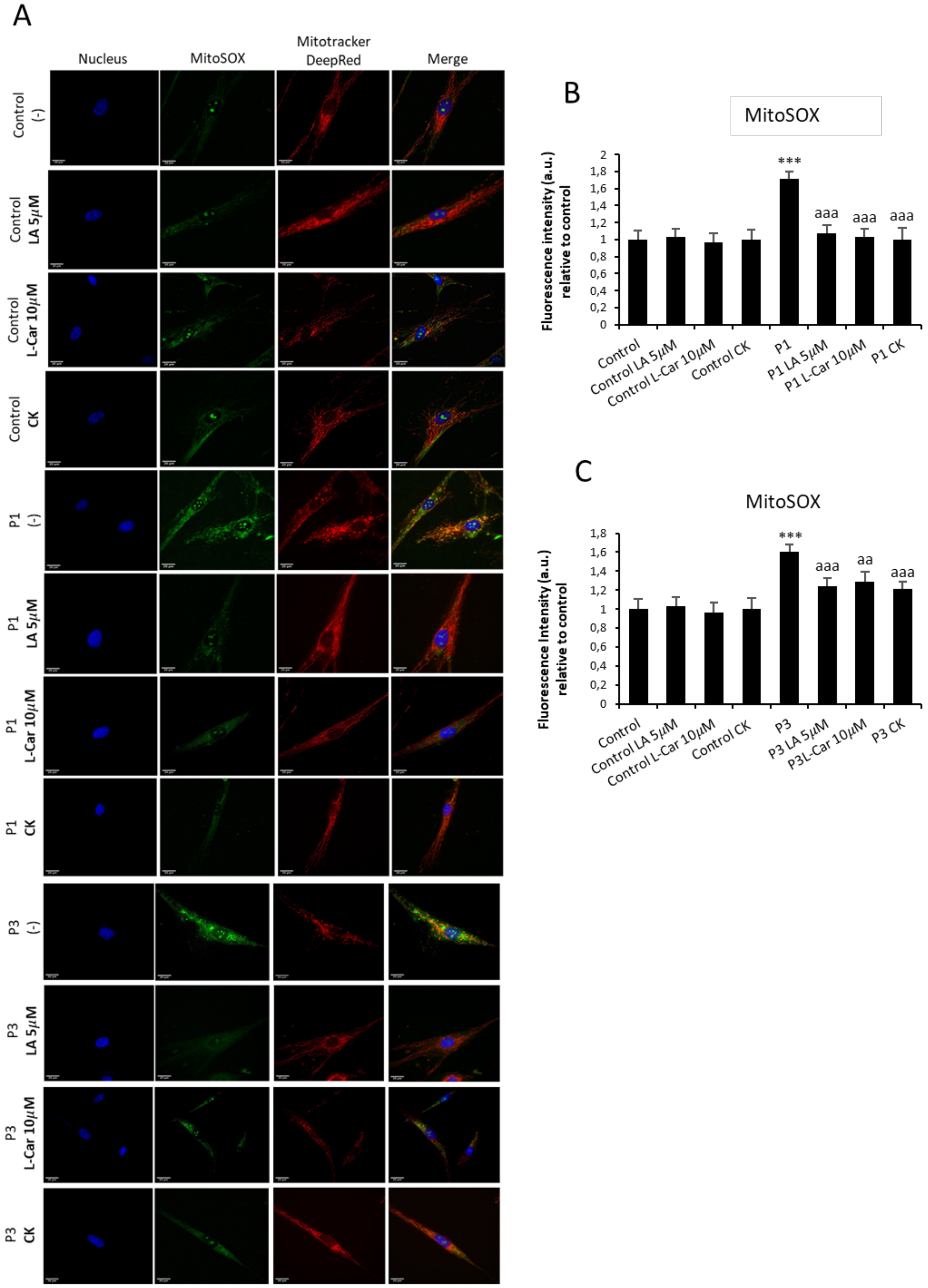

Supplementary Figure S12. Measurement of mitochondrial reactive oxygen species (ROS) generation in treated and untreated Control and NM cells (P1 and P3). Control and NM

fibroblasts (P1 and P3) were treated with 5 mM LA and 10 mM LCAR individually or in combination for 7 days. **(A)** Representative images of mitochondrial superoxide generation in **treated and untreated Control and NM cells (P1 and P3)** stained with MitoSOX™ Red. Mitochondrial network were revealed by MitoTracker™ DeepRed. Nuclei were revealed by DAPI staining. Images were taken under widefield fluorescence microscope using the 40x lens and processed by the ImageJ software. Scale bar = 20 µm. (**B**) Fluorescence quantification of MitoSOX™ Red signal in patient P1 cells (ACTA1 mutation). **(C)** Fluorescence quantification of MitoSOX™ Red signal in patient P3 cells (NEB mutation). Data represent the mean ± SD of three separate experiments (at least 100 cells for each condition and experiment were analysed). ***p<0.001 between NM cells and controls; $^{aa}p<0.001$, $^{aaa}p<0.001$ between untreated (-) and treated NM.
